# Whole-exome sequencing of primary plasma cell leukemia discloses heterogeneous mutational patterns

**DOI:** 10.18632/oncotarget.4028

**Published:** 2015-05-25

**Authors:** Ingrid Cifola, Marta Lionetti, Eva Pinatel, Katia Todoerti, Eleonora Mangano, Alessandro Pietrelli, Sonia Fabris, Laura Mosca, Vittorio Simeon, Maria Teresa Petrucci, Fortunato Morabito, Massimo Offidani, Francesco Di Raimondo, Antonietta Falcone, Tommaso Caravita, Cristina Battaglia, Gianluca De Bellis, Antonio Palumbo, Pellegrino Musto, Antonino Neri

**Affiliations:** ^1^ Institute for Biomedical Technologies, National Research Council, Milan, Italy; ^2^ Department of Clinical Sciences and Community Health, University of Milan, Milan, Italy; ^3^ Hematology, Foundation IRCCS Ca' Granda Ospedale Maggiore Policlinico, Milan, Italy; ^4^ Laboratory of Pre-Clinical and Translational Research, IRCCS-CROB, Referral Cancer Center of Basilicata, Rionero in Vulture (PZ), Italy; ^5^ Hematology, Department of Cellular Biotechnologies and Hematology, La Sapienza University, Rome, Italy; ^6^ Hematology Unit, Azienda Ospedaliera di Cosenza, Cosenza, Italy; ^7^ Hematologic Clinic, Azienda Ospedaliero-Universitaria Ospedali Riuniti di Ancona, Ancona, Italy; ^8^ Department of Biomedical Sciences, Division of Hematology, Ospedale Ferrarotto, University of Catania, Catania, Italy; ^9^ Hematology Unit, IRCCS “Casa Sollievo della Sofferenza” Hospital, San Giovanni Rotondo, Italy; ^10^ Department of Hematology, Ospedale S. Eugenio, Tor Vergata University, Rome, Italy; ^11^ Department of Medical Biotechnology and Translational Medicine, University of Milan, Milan, Italy; ^12^ Division of Hematology, University of Torino, A.O.U. San Giovanni Battista, Torino, Italy; ^13^ Scientific Direction, IRCCS-CROB, Referral Cancer Center of Basilicata, Rionero in Vulture (PZ), Italy

**Keywords:** whole-exome sequencing, plasma cell leukemia, multiple myeloma, mutations

## Abstract

Primary plasma cell leukemia (pPCL) is a rare and aggressive form of plasma cell dyscrasia and may represent a valid model for high-risk multiple myeloma (MM). To provide novel information concerning the mutational profile of this disease, we performed the whole-exome sequencing of a prospective series of 12 pPCL cases included in a Phase II multicenter clinical trial and previously characterized at clinical and molecular levels. We identified 1, 928 coding somatic non-silent variants on 1, 643 genes, with a mean of 166 variants per sample, and only few variants and genes recurrent in two or more samples. An excess of C > T transitions and the presence of two main mutational signatures (related to APOBEC over-activity and aging) occurring in different translocation groups were observed. We identified 14 candidate cancer driver genes, mainly involved in cell-matrix adhesion, cell cycle, genome stability, RNA metabolism and protein folding. Furthermore, integration of mutation data with copy number alteration profiles evidenced biallelically disrupted genes with potential tumor suppressor functions. Globally, cadherin/Wnt signaling, extracellular matrix and cell cycle checkpoint resulted the most affected functional pathways. Sequencing results were finally combined with gene expression data to better elucidate the biological relevance of mutated genes. This study represents the first whole-exome sequencing screen of pPCL and evidenced a remarkable genetic heterogeneity of mutational patterns. This may provide a contribution to the comprehension of the pathogenetic mechanisms associated with this aggressive form of PC dyscrasia and potentially with high-risk MM.

## INTRODUCTION

Plasma cell leukemia (PCL) is a highly aggressive form of plasma cell (PC) dyscrasia defined by the presence of more than 20% of circulating PCs in peripheral blood and/or an absolute circulating PC count greater than 2 × 10^9^/l [1]. PCL can be classified as either primary (pPCL), originating *de novo* at diagnosis, or secondary (sPCL), as a progression from a previous multiple myeloma (MM). PCLs occur rarely, being observed in approximately 2–4% of PC dyscrasias, with pPCL accounting for approximately 50–70% of all cases [2–4]. PCL prognosis is very poor, with a median survival of 7–13 months for pPCL, and even worse for sPCL (2–7 months) [4, 5]. The pathogenetic mechanisms involved in either the primary or secondary forms remain to be fully elucidated [1, 4, 5].

As demonstrated by us and others, PCL shows more complex and heterogeneous molecular patterns than MM, with a greater number of associated genomic aberrations and, in particular, a higher incidence of 13q and 17p deletions and translocations involving immunoglobulin heavy chain locus, whereas very few patients with hyperdiploidy have been described [3, 6–10]. Overall, these findings also suggest that pPCL may represent a valid model for genetic and molecular alterations associated with high-risk MM.

Recently, we provided an extensive biological and molecular characterization of a panel of 23 pPCLs included in a Phase II prospective trial, aiming to evaluate the efficacy of novel biological drugs in the treatment of this aggressive form [11]. In particular, our data based on FISH and SNP array techniques showed the presence of IgH translocations in 87% of pPCL cases, with prevalence of t(11;14) and t(14;16), the occurrence of chr 13 deletion in 74% and of *TP53* deletion/mutations in approximately 40% of cases, and a low frequency of *BRAF* and *RAS* gene mutations [10]. Furthermore, microarray expression profiling revealed the presence of specific transcriptional signatures compared to MM, identifying genes and miRNAs potentially associated with clinical outcome in pPCL [12, 13].

Recently, next-generation sequencing (NGS) studies of MM enlarged the knowledge about the genetic landscape of this disease. Indeed, they collectively provided remarkable information concerning gene mutation signatures as well as the clonal and subclonal heterogeneous structure at diagnosis and its evolution during disease progression [14–17]. However, to the best of our knowledge, no NGS data are currently available in representative series of PCL. Here, we took advantage of our prospective series to decipher, by means of whole-exome sequencing (WES), the pattern of coding somatic mutations in pPCL. Sequencing results were combined with copy number and gene expression data to achieve a comprehensive and integrated view of the molecular landscape of pPCL.

## RESULTS

### Identification of coding somatic variants in pPCL

WES of 12 highly purified PC samples, together with five matched normal controls, was performed by Illumina technology. A mean of 95 M raw reads was generated per sample, of which 98% uniquely mapped to the GRCh37 reference genome. Referring to captured regions (62 Mb), we achieved a target coverage of 97% and a mean depth of 42.3x for both tumor and control cases (Suppl Table S1).

Since matched controls were not available in all pPCL cases, traditional somatic callers such as Mutect could not be used in our study. Therefore, to analyze all the 12 samples using the same method, we implemented a custom pipeline based on GATK caller using at first public databases and then the pool of control samples to filter out germline variants. Following this approach, we initially identified more than 52, 000 raw variants per tumor sample, including both single nucleotide variants (SNVs) and insertions/deletions (indels) (Suppl Table S2). Since our primary interest was in coding regions and splice sites, we applied a series of stringent filters to select coding non-silent variants, including non-synonymous (non-syn) SNVs and indels. First of all, after evaluation of target coverage in relation to sequencing depth, we chose to set at 10x the threshold to reduce the false positive rate. At this depth, all samples (both tumor and control cases) had at least 85% of covered target (Suppl Figure S1). Then, we removed all positions mapping out of coding regions and all variants already annotated in public databases, unless the same variant was also included in COSMIC or NCBI ClinVar catalogues (see Materials and Methods). Thus, we obtained a mean of 1, 525 coding non-syn SNVs and 130 coding indels per tumor sample (Suppl Table S2). The CD138-negative bone marrow populations, available for five patients, were used at this step to filter out germline background, thus obtaining a mean of 149 and 9 coding somatic non-syn SNVs and indels per sample, respectively (Table 1). Lastly, we addressed specific analyses to the identification of somatic sub-clonal variants in the five tumor/normal pairs, and we found 93 sub-clonal non-syn SNVs and 1 sub-clonal insertion in 94 genes (Table 1). All but one (in *TCHH* gene) were private variants occurring in individual cases (details in Suppl File S1). Full description of bioinformatics pipeline is provided in Supplementary Methods.

**Table 1:** Coding somatic non-silent variants identified in pPCL panel

Sample	Somatic		Somatic sub-clonal		Total No. Non-syn SNVs	Total No. Indels	Total No. Somatic Variants
	Non-syn SNVs	Indels	Non-syn SNVs	Indels			
PCL-016	148	4	17	0	165	4	169
PCL-017	160	11	0	0	160	11	171
PCL-018	25	2	9	1	34	3	37
PCL-019	133	5	17	0	150	5	155
PCL-020	195	15	0	0	195	15	210
PCL-026	232	6	34	0	266	6	272
PCL-027	156	16	0	0	156	16	172
PCL-030	179	11	0	0	179	11	190
PCL-032	176	8	0	0	176	8	184
PCL-035	203	14	0	0	203	14	217
PCL-036	146	16	0	0	146	16	162
PCL-038	40	2	17	0	57	2	59

**Figure 1:**
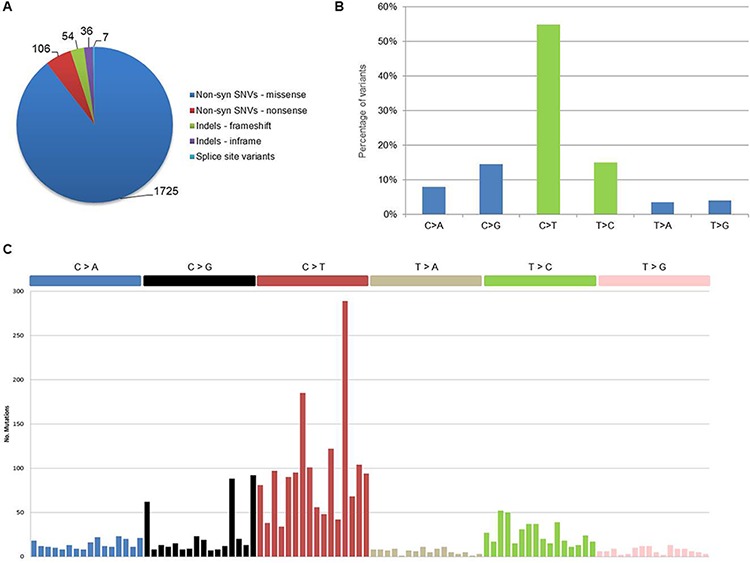
Coding somatic variants identified in the 12 pPCL samples **A.** Distribution of coding somatic non-silent variants according to amino acid changes or reading frameshifts. **B.** Classification of coding somatic SNVs (both syn and non-syn) by nucleotide change. Transitions are in green (C > T, T > C) and transversions are in blue (C > A, C > G, T > A, T > G). **C.** Mutational signatures in pPCL. Coding somatic SNVs identified in the 12 samples were classified according to nucleotide change and sequence context into the 96 possible mutated trinucleotides (listed horizontally according to the conventional order). Substitutions are displayed in different colors along the horizontal axis and number of mutations attributed to each type is shown on the vertical axis.

Globally, we identified a total of 1, 928 coding somatic non-silent variants (mean 166 per sample), including 1, 831 non-syn SNVs (missense and nonsense), 90 indels and 7 splice site variants, distributed on 1, 643 protein-coding genes (Figure 1A). All variants are provided with full annotation in Supplementary File S1 and summarized by gene in Supplementary File S2. Eighty-one variants were validated by independent sequencing experiments, with a confirmation rate of 95%, in line with other cancer genome sequencing studies (Suppl File S3).

Concerning nucleotide changes, we observed an excess of C > T transitions, in agreement with what recently reported in MM [16] (Figure 1B). Furthermore, when looking at nucleotide context for coding somatic mutations in the whole dataset, we observed the occurrence of two main mutational signatures (Figure 1C). The first signature (corresponding to Signature 1A, according to the current classification [18]) was dominated by C > T transitions at NpCpG sites resulting from the spontaneous deamination of 5-methyl cytosine associated with aging. The second signature (Signature 2) was characterized by the prevalence of C > T and C > G in a TpCpN context, resulting from the over-activity of APOBEC cytidine deaminase enzymes [18]. Both signatures were recently described in MM [16] as well as in other cancer types [18]. Evaluating the contribution of each nucleotide context in individual pPCL samples, few cases appeared driven by one single mutational signature, while the majority showed an admixture of the two processes (Suppl Figure S2). In line with recently published data, this seems to be a common feature in cancer genomes, regardless of tumor type [18]. Interestingly, when considering the translocation groups of our dataset, three out of the four samples carrying the t(14;16) translocation involving *MAF* gene showed a distinct APOBEC mutational signature, while among the other five samples carrying t(11;14) or t(4;14), one case presented a distinct age-related signature and three other samples showed an admixture of both Signatures 1A and 2, with the predominant contribution of age-related process. Thus, in agreement with what recently presented for MM by Walker et al. [19], we can confirm also for pPCL (although in a relatively small dataset) that t(14;16) cases predominantly had an APOBEC signature.

### Integration with copy number alterations

**Figure 2:**
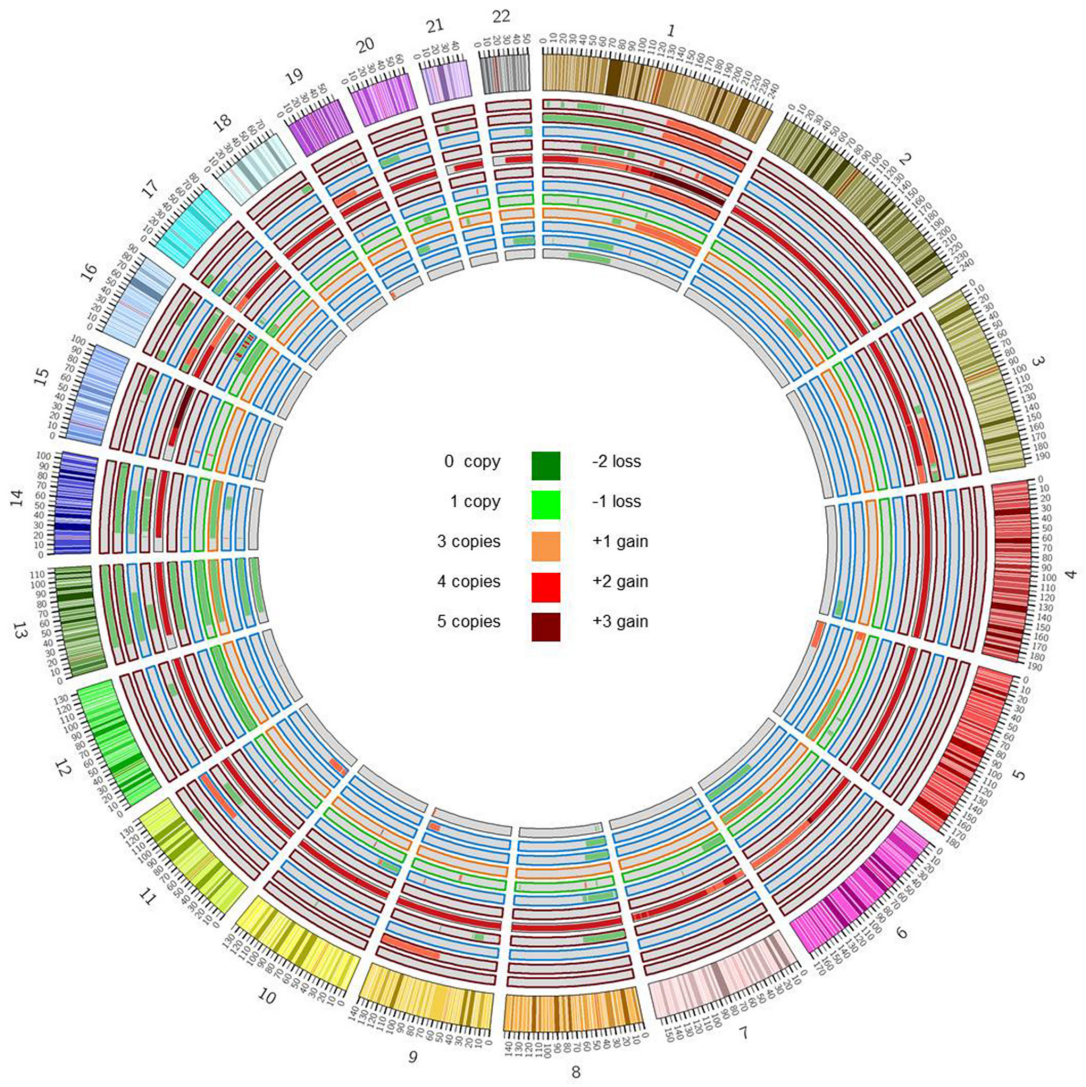
Copy number analysis Copy number alterations were identified starting from WES data by using EXCAVATOR software. The Circos plot represents somatic amplifications (red) and deletions (green) found in each case, distributed across all chromosomes. Samples are displayed according to increasing case number from outer to inner track. Colors of track borders indicate translocation groups: t(14;16) in red, t(11;14) in blue, t(14;20) in green, t(4;14) in orange. PCL-020 resulted as outlier with the greatest number of CNAs and a basal tetraploidy with regions till to five copies.

**Figure 3:**
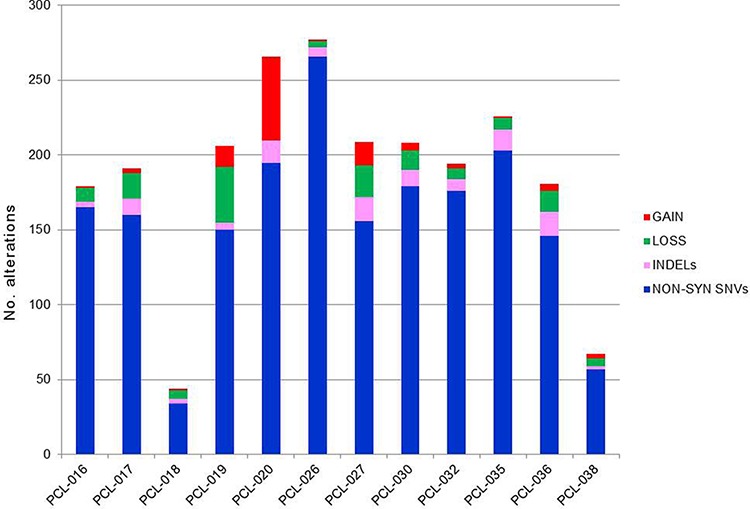
Global amount of somatic alterations per sample The numbers of coding somatic non-silent variants (non-synonymous SNVs and indels) and CNA regions (gains and losses) were summed up by sample, to have an overview of the global burden of somatic alterations carried by each case.

Somatic copy number alterations (CNAs) in the 12 pPCLs were identified from WES data using EXCAVATOR software. Globally, we found a mean of 20 CNA traits per sample, with more frequent deletions than amplifications (Figure 2; Suppl File S4). The CNAs here identified were concordant to those previously characterized in the same samples by using Affymetrix SNP Array [10] and in agreement with the known PCL genomic signature, including deletions of chrs 1p, 8p, 13q and 17p, and gain of chr 1q. To evaluate the global amount of somatic alterations carried by each of the 12 pPCLs, coding somatic variants (non-syn SNVs and indels) and CNA results were summed up by sample (Figure 3). PCL-026 and PCL-020 resulted the most impacted samples, with the highest number of variants and CNAs, respectively.

Lastly, in order to assess CN state of the 1, 643 mutated genes, WES and CNA results were integrated for all the 12 pPCLs (Suppl File S5). We identified three homozygous deletions involving three genes, namely *BIRC2* on chr 11 and *TRPM6* on chr 9 (both in PCL-019) and *TRAF3* on chr 14 (in PCL-017). Furthermore, we found 77 genes concomitantly affected by mutation and 1-copy loss, suggesting a potential tumor suppressor gene (TSG) function (Suppl File S5). Among them, *TP53* resulted the most recurrently disrupted gene (three missense variants and one frameshift indel in four cases, all associated with deletion of the other allele (see below for further details)), while *BIRC2* and *TRAF3*, besides the homozygous deletions above described, were found biallelically affected in two additional cases, presenting respectively a truncating mutation and a frameshift deletion, both associated with loss of the remaining allele.

### Candidate driver genes in pPCL

Using DOTS-Finder tool and taking into account gene mutation rates calculated in our samples with respect to COSMIC and TCGA databases, we identified 14 statistically significant recurrently affected genes with potential driver role in pPCL. Among them, we found genes involved in cell-matrix adhesion and membrane organization (*SPTB, CELA1*), cell cycle and apoptosis (*CIDEC*), genome stability (*KIF2B*), RNA binding and degradation (*DIS3, RPL17*), and protein folding (*CMYA5*) (Table 2). In particular, *DIS3*, coding for an RNA exonuclease catalytic subunit of the exosome complex and described as one of the most recurrently mutated genes in MM [14, 17, 20], resulted affected in three PCLs (25%). In two samples the mutation involved the same aminoacid position but introducing two different changes (R780K and R780T), both already reported in literature for MM [17, 20]; in the third case, the mutation was in homozygous state, associated with deletion of the other allele. In addition, the gene showed hemizygous deletion in other seven pPCLs. Although deserving investigations in larger collections to assess their actual recurrence, these genes might represent novel potential markers for translational clinical applications.

**Table 2:** Statistically significant recurrently affected genes with potential driver role in pPCL

Gene	Gene Name	No. Cases (%)	No. Variants	No. Non-syn SNVs	No. Indels	*q*-value	Function
HLA-DQA1	major histocompatibility complex, class II, DQ alpha 1	3 (25%)	3	-	3*	3.89E-09	immune response
CIDEC	cell death-inducing DFFA-like effector c	3 (25%)	1	1*	-	3.07E-05	apoptosis
CELA1	chymotrypsin-like elastase family, member 1	3 (25%)	1	-	1	7.42E-05	proteolysis, cell membrane and matrix organization
SRRM5	serine/arginine repetitive matrix 5	3 (25%)	2	2*	-	7.89E-05	unknown
CCDC144NL	coiled-coil domain containing 144 family, N-terminal like	3 (25%)	2	-	2	8.59E-05	unknown
SPTB	spectrin, beta, erythrocytic	3 (25%)	5	5	-	0.00280	cell membrane organization and stability
DIS3	exosome catalytic subunit DIS3	3 (25%)	3	3	-	0.00427	RNA binding, RNA degradation
FAM166B	family with sequence similarity 166, member B	2 (16%)	1	-	1*	0.01062	unknown
RPL17	ribosomal protein L17	2 (16%)	2	1	1	0.01405	RNA binding, translation
CMYA5	cardiomyopathy associated 5	1 (8%)	7	7	-	0.02032	protein binding
UNC80	unc-80 homolog	4 (33%)	4	4	-	0.02762	ion transmembrane transport
SCN9A	sodium channel, voltage-gated, type IX, alpha subunit	3 (25%)	3	3	-	0.03008	ion transmembrane transport
ZNF598	zinc finger protein 598	3 (25%)	1	-	1*	0.03008	translation repressor
KIF2B	kinesin family member 2B	2 (16%)	2	1	1	0.09976	chromosome segregation, genome stability

Note:

* Identical recurrent variants found in unpaired pPCL samples annotated in both dbSNP and COSMIC catalogue and confirmed somatic in COSMIC website.

### Pathways affected by somatic mutations in pPCL

After removal of potentially spurious genes frequently mutated in cancer but not necessarily relevant to tumor biology [21], functional enrichment analysis of the mutated genes using ToppGene evidenced five significantly enriched pathways, including cadherin and Wnt signaling, extracellular matrix (ECM)-receptor interaction, ECM organization, and G2/M cell cycle checkpoint (Table 3).

**Table 3:** Pathways significantly enriched in mutated genes by ToppGene analysis

Pathway	Source	*p*-value	FDR *q*-value	No. Damaging/Total variants	Genes
Cadherin signaling pathway	PantherDB (ID P00012)	1.26E-07	3.07E-04	24/48	PCDH15*, FZD6, PCDH7*, PCDH20*, CDH20*, DCHS1*, CDH17*, CDH4*, CDH9*, CDH23*, CTNNA2, PCDHGB1*, PCDHGC5*, PCDHGC4*, PCDHGA2*, PCDHGA1*, PCDHA7*, PCDHA13*, PCDHAC1*, PCDHB2*, PCDHB3*, PCDHB7*, PCDHB8*, PCDHA2*, PCDHA1*, PCDHA3*, CELSR3*, FAT1*, FAT2*, FER, FZD10, CDHR2*, FAT3*, YES1, PCDH11X*
ECM-receptor interaction	BioSystems: KEGG (ID 83068)	2.01E-06	2.45E-03	9/26	CD36, SV2B, COL1A2^†^, COL1A1^†^, COL5A1^†^, COL6A3^†^, COL4A2^†^, DAG1^†^, COL6A6^†^, RELN^†^, TNN^†^, FN1^†^, THBS3, TNXB^†^, HMMR, HSPG2^†^, ITGA1, LAMB1^†^, LAMA4^†^, LAMA5^†^, LAMA2^†^, LAMA3^†^
Cell Cycle G2/M Checkpoint	MSigDB C2: BioCarta (ID M8560)	1.25E-05	1.02E-02	13/20^‡^	ATM, ATR, BRCA1, CDC25A, CDKN1A, PRKDC, EP300, CHEK2, RPS6KA1, TP53
Wnt signaling pathway	PantherDB (ID P00057)	2.17E-05	1.18E-02	36/66^‡^	MYH13, PCDH15*, FZD6, PCDH7*, MYH7, PCDH20*, CDH20*, DCHS1*, CDH17*, CDH4*, CDH9*, CDH23*, PRKCZ, PPP2R5E, PPP3R2, CREBBP, CTNNA2, PLCB4, PCDHGB1*, PCDHGC5*, PCDHGC4*, PCDHGA2*, PCDHGA1*, DVL3, PCDHA7*, PCDHA13*, PCDHAC1*, PCDHB2*, PCDHB3*, PCDHB7*, PCDHB8*, PCDHA2*, PCDHA1*, PCDHA3*, EP300, CELSR3*, FAT1*, FAT2*, SRCAP, FZD10, INO80, TP53, CDHR2*, FAT3*, PCDH11X*, KREMEN1, ITPR2, MYH14, PLCB1
Extracellular matrix organization	BioSystems: REACTOME (ID 576262)	2.50E-05	1.18E-02	22/51	ACTN1, ACAN^†^, COL6A5^†^, DDR2, LTBP4^†^, ADAMTS9^†^, COL1A2^†^, COL1A1^†^, COL15A1^†^, COL17A1^†^, COL9A3^†^, PDGFB, COL9A1^†^, COL5A1^†^, COL6A3^†^, COL4A2^†^, PLEC, VCAN^†^, DAG1^†^, ADAMTS16^†^, ADAMTS18^†^, COL6A6^†^, BCAN^†^, PSEN1, DSPP^†^, TNN^†^, PTPRS, FBN2^†^, FGA, FN1^†^, ADAMTS5^†^, TNXB^†^, ADAM18, LEPREL1^†^, HSPG2^†^, ICAM4, ITGA1, LOXL4, LAMB1^†^, LAMA4^†^, LAMA5^†^, LAMA2^†^, LAMA3^†^, FBN3^†^

Note:

* 30 genes showing a cadherin domain according to significant enrichment by ToppGene analysis (InterPro IPR002126 domain, *q*-value 3.24E-08, FDR correction).

† 34 genes annotated as proteinaceous extracellular matrix (ECM) cellular components according to significant enrichment by ToppGene analysis (GO Cellular Component GO:0005578, *q*-value 4.62E-04, FDR correction).

‡ Significant enrichment for damaging variants (*q*-value < 0.05, Bonferroni correction).

In cadherin/Wnt signaling pathways, we globally recognized 51 genes, 30 of which (59%) showed a cadherin domain. Among cadherin genes, the most recurrent ones were *PCDH15* (four cases), *DCHS1* and *FAT3* (each in three samples). Ten pPCLs showed mutations in two or more cadherin genes. Furthermore, cadherin genes were principally identified in the protocadherin (PCDH) family (19/30, 63%). Most *PCDH* mutated genes (15/19, 79%) belonged to the three tandem 5q31 region gene clusters [22].

Notably, 49 genes were involved in ECM-receptor interaction and organization pathways, 34 of which (69%) annotated as proteinaceous ECM cellular components, including collagen and laminin genes, ADAM metallopeptidases and members of the aggrecan/versican proteoglycan family. Ten PCLs resulted affected in these ECM cellular components, and six cases presented mutations in five genes or more.

Finally, 10 genes were involved in G2/M cell cycle checkpoint pathway in nine PCLs. Among them, *TP53* and *ATM* (four cases) were the most recurrent ones, followed by *ATR* and *CDKN1A* (three cases) and *BRCA1* (two cases). Notably, six PCL cases showed the involvement of the kinase *ATM* (PCL-017, PCL-026, PCL-030) or *ATR* (PCL-019, PCL-035) in a mutually exclusive fashion, or in a combined manner (PCL-020). Additionally, *TP53* gene was frequently found altered in association with at least one another gene of this pathway: *ATM* (PCL-017), *CDKN1A* and *RPS6KA1* (PCL-027), or both *ATM* and *CDKN1A*, together with *CDC25A, CHEK2* and *PRKDC* genes, in the most affected case (PCL-030). No concurrent mutations in *ATR* and *TP53* were evidenced.

Furthermore, we identified other eight affected pathways that, although did not reach statistical significance after *p*-value correction, are noteworthy since notoriously involved in MM and lymphoid malignancies [23, 24] and targeted by current molecular therapies [25]. They included the proteasome and ubiquitin-mediated proteolysis machineries and the TNF, NF-kB, PI3k-Akt, MAPK and Hippo signaling pathways (Suppl Table S3).

Interestingly, if looking at impact prediction of variants affecting all these pathways, we observed a statistically significant enrichment for damaging variants (*P* < 0.01, Fisher exact test) (Suppl Figure S3A). The probability of having the same fraction of damaging variants randomly sampling the same number of mutations in our dataset was estimated to be 10^−5^ and 10^−4^ for the five enriched and the eight selected pathways, respectively (Suppl Figure S3B–S3C). Moreover, when tested individually, Wnt signaling and G2/M cell cycle checkpoint (*q*-value < 0.05) and MAPK pathway (*q*-value < 0.01) maintained a significant enrichment for damaging variants with respect to background.

### Comparison with multiple myeloma recurrent genes

**Figure 4:**
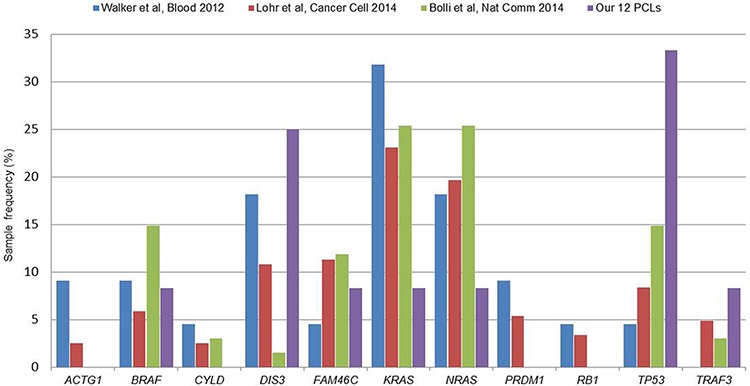
Recurrence of the 11 multiple myeloma genes in our pPCL series with respect to MM published datasets Recently published datasets for multiple myeloma include: Walker *et al*., Blood 2012 (22 cases, [20]), Lohr *et al*., Cancer Cell 2014 (203 cases, [17]) and Bolli *et al*., Nat. Commun. 2014 (67 cases, [16]). Recurrence rates were calculated as the percentage of positive samples on the total number of cases included in each study.

We evaluated in our series the occurrence of somatic variants in 11 genes recurrently mutated in MM [17]. Globally, seven genes were affected in our samples (*TP53, DIS3, BRAF, KRAS, NRAS, FAM46C, TRAF3*), while *ACTG1, CYLD, PRDM1* and *RB1* carried no variant (Suppl Table S4). *TP53* was the most frequent, completely inactivated in four pPCLs (33%) as above mentioned. Three pPCLs (25%) carried variants in *DIS3*, in addition to other seven samples showing hemizygous deletion, and this gene resulted a statistically significant driver gene in our dataset. The remaining five genes showed individual missense and frameshift variants. *BRAF* carried two missense mutations in the same sample, both already described in MM. *FAM46C* presented a novel variant, never previously reported for other tumors, besides being deleted in other five PCLs. *KRAS* and *NRAS* resulted affected in two distinct samples and both mutations were already described for MM; moreover, *NRAS* resulted deleted in five PCLs, while amplified only in the sample harboring the mutation. Finally, *TRAF3* was completely inactivated in two samples, as aforementioned. All variants were confirmed by Sanger sequencing (Suppl File S3). Overall, we observed that *KRAS* and *NRAS* were three-fold less frequent, while *DIS3* and *TP53* were till to two-fold more recurrent in pPCL than MM considered as a whole [16, 17, 20] (Figure 4).

### Gene expression profiles of mutated genes in MMs and pPCLs

**Figure 5:**
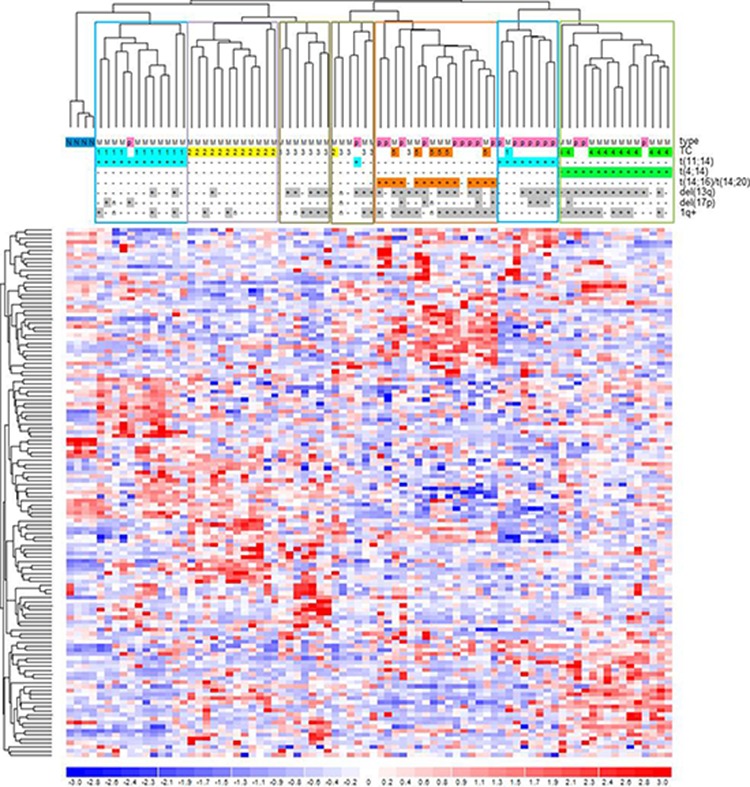
Hierarchical clustering of the 55 MM-21pPCL-4N dataset based on the expression levels of the 132 most variable and mutated genes Data were previously generated on GeneChip Human Gene 1.0 ST array platform [12]. Samples were labelled according to tumor type (MM, multiple myeloma; p, primary plasma cell leukemia), translocation/cyclin D (TC) classification and the main associated cytogenetic alterations. Significant sample clusters, according to major IgH translocations, are highlighted.

A proprietary dataset of 55 MM and 21 pPCL cases (comprising the 12 in this study) and four bone marrow healthy donors profiled on Affymetrix Gene 1.0 array [12] was exploited to assess the expression profiles of the mutated genes (1, 598 of which included in the chip array). A hierarchical clustering based on the 132 mutated genes found among the most variably expressed genes (2AVEFC) of our dataset revealed that sample grouping was principally driven by the main cytogenetic translocations (Figure 5). Specifically, we found a good separation of the two t(4;14) (TC4) and t(14;16)/t(14;20) (TC5) MM-PCL sample groups (15/15 and 14/14, respectively; *P* < 10^−4^), whereas t(11;14) cases (TC1) were divided in two sub-branches, clearly separating MM from PCL samples (11/12 MMs and 7/8 PCLs; *P* < 10^−4^). Additionally, three distinct clusters were identified for TC2 (12/13; *P* < 3 × 10^−4^) and TC3 MM cases (7/12; *P* < 10^−4^, and 4/12; *P* < 9.4 × 10^−3^). Notably, functional enrichment in regulation of cellular developmental process and morphogenesis, phosphatase activity, ECM/cytoskeleton organization, cell adhesion and endocytosis processes was observed in these 132 genes (Suppl Table S5). Interestingly, the probability of replicating the same sample clustering using a comparable number of genes randomly extracted from the whole 2AVEFC list (regardless of mutational state) was calculated as *P* < 0.004. This finding indicated that these 132 genes, representing the 11% of the 2AVEFC list, had peculiar clustering abilities and strongly suggested that genes found mutated in pPCL may also have relevant biological roles in the context of the different molecular and clinical types of MM.

Finally, a two-class supervised analysis comparing pPCL to MM samples on the 1, 598 mutated genes identified 102 differentially expressed genes (DEGs), of which 42 up- and 60 down-regulated in pPCL versus MM group (Suppl Table S6). Specifically, up-regulated genes were functionally enriched in regulation of transcriptional processes, while several down-regulated genes were involved in cell death regulation. Interestingly, concerning genes included in the five enriched functional pathways aforementioned, we found *ATM* and *CHEK2* (G2/M cell cycle checkpoint) as well as *CD36, HMMR* and *RELN* (ECM-receptor interaction pathway) as down-regulated in pPCL versus MM, whereas *EP300* (Wnt signaling pathway) resulted up-regulated in pPCL as compared to MM samples. Notably, 33 out of the 102 mutated DEGs (32%) were in common with our published 503-gene transcriptional signature distinguishing pPCL and MM [12].

## DISCUSSION

Over the last few years, NGS technology has extended our knowledge of the genetic alterations associated with transformation and progression in MM. Notably, these studies showed a considerable number of somatic variants per patient, few recurrently mutated genes with pathogenetic significance, and an heterogeneous sub-clonal structure at presentation [16, 17, 20, 26]. Furthermore, although performed on only two PCL cases, Walker et al. [15] recently demonstrated that number and pattern of somatic variants at PCL stage are higher and more complex than in MM, smoldering multiple myeloma (SMM) and monoclonal gammopathy of undetermined significance (MGUS). In the present study, we aimed to provide novel and more comprehensive information concerning the mutational profile of pPCL by performing WES of highly purified malignant PCs from a prospective panel of pPCL patients already extensively characterized at clinical and molecular levels in our previousreports [10–13].

Our WES analysis in pPCL confirmed and extended the remarkable genetic heterogeneity associated with MM. Based on our results, 1, 928 coding somatic non-silent variants distributed on 1, 643 genes were identified in the whole dataset, with a mean of 166 variants per sample. However, as already observed for MM, the recurrence of identical variants did not appear a frequent event in pPCL, where only 1.9% of variants were recurrent in two or more samples, even if affecting genes with pathogenetic relevance already reported in COSMIC and MM [14]. Though we could have slightly over-estimated the number of identified variants due to the availability of paired samples only in five patients, our study represents so far the most comprehensive overview of the mutational patterns of primary PCL.

In our study on pPCL, we observed that, among those genes found recurrently mutated in MM, *KRAS* and *NRAS* were three-fold less frequent, while *DIS3* and *TP53* were till to two-fold more recurrent in pPCL than MM considered as a whole [16, 17, 20]. These differences in mutational frequencies could be partially explained by the composition of our pPCL series in terms of cytogenetic abnormalities, not strictly comparable to that generally observed for MM. This mainly concerns *TP53* and *DIS3*, whose mutations have been reported in several MM series as preferentially associated, respectively, with chr 17p deletion [16, 27] and non-hyperdiploid status [17]. Both these conditions are over-represented in our pPCL dataset, in line with previous reports in other pPCL series [3, 9, 28]. In particular, as regards *TP53* mutations, it should be noted, as aforementioned, that all the four *TP53*-mutated cases also carried del(17p), and the frequent co-occurrence of these two genetic alterations is compatible with the demonstrated association of *TP53* inactivation with advanced forms of malignancy (also substantiated by its nearly ubiquity in MM cell lines) [29]. Three of the four *TP53* mutations we found (I195T, R273C, P278L) were deleterious missense variants targeting the DNA binding domain and frequently reported as mutated (IARC TP53 Database, http://p53.iarc.fr/), also in MM patients [16, 17]; the fourth variant was a deletion of ten nucleotides, of which eight at the 3′-end of intron 9 and two at the 5′-end of the following exon 10, introducing a reading frameshift and consequently a premature stop codon in the first part of the oligomerization domain (I332Pfs*4). As regards *NRAS* and *KRAS*, a slight enrichment of mutated cases among hyperdiploid MM patients can be observed in both the main WES studies for MM: both *NRAS* and *KRAS* were reported mutated in 18.5% of non-hyperdiploid (NHD) and 30% of hyperdiploid (HD) patients in the dataset from Bolli et al. [16], while in the cohort by Lohr et al. [17] *NRAS* mutations were detected in 16.3% of NHD and 22.4% of HD patients, and *KRAS* mutations in 18.6% of NHD and 26.7% of HD cases (although association did not reach statistical significance). Based on these data, the low occurrence of *NRAS* and *KRAS* mutations in our 12 pPCLs could be partly expected. Concerning *DIS3*, although its role in the pathogenesis of the disease remains to be elucidated, this gene was characterized as potential tumor suppressor in MM. This suggestion is based on the loss of enzymatic activity caused by the MM-associated *DIS3* mutations that have been functionally studied [30], and on the observation of frequent loss of heterozygosity involving *DIS3* (also occurring in our dataset) due to the combination of gene mutation and chr 13 deletion. Anyhow, further investigations in considerably larger cohorts will be definitely useful to confirm the involvement of these genes in pPCL and their actual recurrence with respect to MM.

Interestingly, we identified significantly enriched pathways associated with mutated genes in pPCL, namely cadherin/Wnt signaling, ECM-receptor interaction and organization, and G2/M cell cycle checkpoint. Notably, both Wnt signaling and G2/M cell cycle checkpoint pathways resulted enriched also in damaging variants, thus enforcing the hypothesis of their crucial involvement in pPCL biology. Finally, several of the candidate driver genes found in our study were included or strictly correlated with these pathways.

Cadherins play a major role in cell-cell adhesion and tissue integrity and homeostasis maintenance and their alterations are associated with tumorigenesis and metastasis; in particular, several protocadherins were recognized as candidate TSGs, mainly silenced by methylation in different solid tumors [31]. Furthermore, cadherins are functionally associated with cytoplasmic members of the catenin family, including β-catenin which represents a crucial player in Wnt signaling [32]. Notably, two genes of the cadherin/Wnt cascade, *PPP2R5E* and *EP300*, resulted as potential drivers for pPCL (though with *q*-value slightly above significance threshold, probably due to the small size of our dataset). *PPP2R5E*, involved in apoptosis control, was proposed as TSG in acute myeloid leukemia [33]. *EP300* encodes a histone acetyltransferase, whose altered transcriptional and epigenetic functions were associated to leukemia and other cancers [34]. Notably, EP300/β-catenin interaction is critical in Wnt pathway regulation and particularly in mediating neoplastic transformation [35]. Moreover, among the mutated cadherin genes interacting with Wnt pathway, *FAT1* (FAT atypical cadherin 1) was found mutated in solid tumors [36] and chronic lymphocytic leukemia [37]. When inactivated, *FAT1* is unable to sequester β-catenin at the cell membrane, which in turn promotes the activation of Wnt cascade and tumor growth [38]. Notably, other *FAT* genes frequently mutated in solid tumors [38] resulted affected in our series, such as *FAT2* (one case) and *FAT3* (three cases), in a mutually exclusive fashion. Interestingly, *FAT3* was found recurrently mutated in a recently published MM series [16].

ECM is part of the bone marrow (BM) microenvironment and includes many components as fibronectin, laminins, collagens and proteoglycans. ECM can provide a protective environment against drug effects, thus favoring the emergence of chemoresistant cancer cells [39]; for instance, fibronectin was found directly involved in the mechanisms of cell adhesion-mediated drug resistance of MM cells [40], whereas collagen degradation in the BM ECM by matrix metalloproteinases may contribute to MM progression [41]. Recent evidences suggested that ECM components expressed by myeloma patients differ from those of healthy individuals, involving the progressive up-regulation of specific ECM proteins in the transition from MGUS to MM [42]. Here, we found several mutated ECM components, including proteoglycans and proteases, involved in ECM homeostasis and remodeling, as well as collagen and laminin genes, which can interact with surrounding cells through integrin membrane receptors and participate in differentiation, adhesion and migration [43]. Finally, enrichment in ECM organization and cell adhesion functions was found in the 132 most variably expressed among the mutated genes in our analyses, further supporting the hypothesis that they could play important pathogenetic roles in these PC disorders. Overall, based on these considerations, alterations of both these pathways may be strongly involved in the mechanisms of extramedullary spread associated with pPCL.

The G2/M cell cycle checkpoint pathway represents a complex process interconnected with several other cascades, first of all with the DNA damage response (DDR) mechanisms. MM and in particular PCL are disorders characterized by high genetic instability; the expression of genes involved in distinct DNA repair mechanisms has recently been associated with a disease-specific prognostic relevance in MM [44]. Here, we evidenced the marked involvement of *TP53* gene. Although referring to a relatively small series, its occurrence was higher than that reported for MM in previous NGS studies [16, 17, 20]. The increased frequency of *TP53* deletions found in our pPCLs with respect to MM is confirmed in all published pPCL series [3, 6, 7, 9, 28] and can be partially explained by the different composition in terms of cytogenetic alterations between pPCL and MM. Furthermore, we reported the recurrent involvement of *ATM* and *ATR* genes, which encode the two checkpoint kinases required for cell cycle arrest and DNA damage repair activation. In particular, the PI3/PI4-kinase *ATR*, carrying four missense variants in three pPCLs, resulted a potential driver gene in our series. Recently, ATR inhibitors showed to sensitize tumor cells to topoisomerase inhibitors already used in clinical trials, thus suggesting the possibility of a combined strategy to enhance cancer treatment efficacy [45]. The co-occurrence of mutations in different members of the DDR cascade downstream to *ATM* or *ATR* (*CHEK2, CDC25A, TP53, CDKN1A, BRCA1*) may suggest a potential synergic role in deregulated DNA repair function in pPCL. This is of particular relevance since our study involved patients that did not receive any previous treatment, suggesting that alterations of this function may represent a specific hallmark of tumorigenesis in pPCL.

Overall, our results suggested a peculiar involvement for a variegated set of genes and pathways in pPCL biology and evidenced the importance of their comprehensive characterization to extend the knowledge of molecular mechanisms responsible for this disease. Moreover, since pPCL is a highly aggressive form of PC dyscrasia, understanding its molecular processes might also shed new light and help to unravel the genetic complexity of high-risk MM. Candidate genes identified in these pathways might be useful as actionable targets for therapeutic approaches, thus opening new perspectives or improving the efficacy of already existing strategies.

## MATERIALS AND METHODS

### Patients and sample preparation

Pathological specimens were collected from 12 untreated pPCL patients, all but one (PCL-038) included in a multicenter Italian clinical trial (RV-PCL-PI-350, EudraCT N° 2008-003246-28), an open label, exploratory, single arm, two-stage study aimed at evaluating safety and anti-tumor activity of combined lenalidomide and dexamethasone as first-line treatment for previously untreated pPCLs [11]. All cases have been characterized by fluorescent in situ hybridization (FISH) for the main MM genomic aberrations (Suppl Table S7) and profiled for whole-genome DNA copy number and gene and miRNA expression by microarray technology [10, 12, 13]. Data are available at NCBI GEO repository (Accession numbers GSE39383, GSE39925 and GSE37053).

Highly purified (≥ 90%) bone marrow PCs were obtained as previously reported [46]. CD138-negative bone marrow population (CD138+ < 0.5%, as assessed by FACS analysis) was obtained for five of the 12 patients and used as normal counterpart for WES.

### Whole-exome capture and sequencing

Whole-exome capture for the 12 pPCLs and five control samples was performed using the TruSeq Exome Enrichment kit (62 Mb) (Illumina, San Diego, CA, USA). Libraries were sequenced on an Illumina GAIIx platform, in paired-end 85-cycle runs (see Supplementary Methods for details). Raw data have been deposited in NCBI Sequence Read Archive (SRA), under Study Accession Number SRP051153.

### WES data analysis

Since matched controls were not available in all pPCL cases, traditional somatic callers could not be used in our study. Therefore, to analyze all the 12 samples using the same method, we implemented a custom pipeline based on GATK UnifiedGenotyper caller and used at first public databases to filter out known polymorphisms and then the pool of control samples to further remove germline variants. Briefly, reads were mapped to the NCBI human reference genome (GRCh37) using BWA [47]. Variant calling for SNVs and indels was performed using GATK software [48], and functional annotation was carried out using Annovar [49], according to Ensembl GenCode v67 gene model. By applying a stringent custom filtering pipeline, any variant already annotated as polymorphism in public databases (dbSNP, 1000 Genomes, ESP) was discarded, unless present also in COSMIC (v67) or NCBI ClinVar database given their potential relevance for tumor or clinical associations. Finally, control samples were used to remove germline background and to select coding somatic non-silent variants, including non-synonymous SNVs and indels. Full description of bioinformatics pipeline and filtering steps is provided in Supplementary Methods.

Confirmation of WES variants was performed by PCR amplification and Sanger or Roche Junior sequencing. Calculation of nucleotide contexts for identification of mutational signatures was performed according to Alexandrov et al. [50].

Since control samples were not available for all the investigated cases, there was the possibility that calls in unpaired samples still included private polymorphisms, not removed because absent in the control pool. To evaluate the possible carry-over of germline background, our pipeline was applied on the five paired pPCLs by analyzing them first in “paired” manner (subtracting to each tumor its matched control, as described in Supplementary Methods) and then in “unpaired” manner (subtracting to each tumor the pool of the other not matched controls). Results showed that the private background was ultimately marginal thanks to the adopted filtering steps (Suppl Table S8). Identical recurrent variants found in two or more samples and annotated in both dbSNP and COSMIC catalogue were manually checked in COSMIC website for somatic status. The few variants (all occurring in unpaired pPCLs) whose somatic status was not confirmed in COSMIC website were flagged for further analyses as they could be residual germline background (Suppl Table S9).

### Copy number analysis and integration

Genome-wide analysis of somatic DNA copy number alterations (CNAs) was performed on WES data using the read count-based EXCAVATOR software [51], with default parameters and setting *c* = 1 (Supplementary Methods). To assess the CN state of mutated genes, genes mapping in CNA regions were retrieved using UCSC RefSeq transcript annotation track. In case of multiple transcripts for the same gene, the largest RefSeq transcript was chosen. Then, this CNA-gene list was intersected with WES mutated gene list, to evaluate the CN state of affected genes across the whole dataset.

### Identification of driver genes

Specifically implemented for the analysis of small tumor datasets, DOTS-Finder tool [52] was applied with default settings to identify significant recurrently affected genes with potential driver role in our series, based on functional and frequentist approaches taking into account gene length, dataset size and number of non-synonymous/synonymous variants found in our samples with respect to COSMIC and The Cancer Genome Atlas (TCGA) cancer databases. Identical recurrent variants found in unpaired pPCL samples and annotated in both dbSNP and COSMIC catalogue but not confirmed as somatic in COSMIC website were considered just once to not over-score corresponding genes. Annovar output was used to produce the initial marf file and a *q*-value < 0.1 (Benjamini-Hochberg correction) was set to define statistically significant driver genes.

### Pathway analysis

Functional enrichment analysis of the mutated genes was performed using ToppGene Suite [53]. Pathways with *q*-value < 0.05 (FDR Benjamini-Hochberg correction) were defined as significantly enriched. Before running ToppGene, a list of potentially spurious genes frequently found mutated in cancer but not necessarily relevant to tumor biology was compiled according to Lawrence et al. [21]. Twenty-three of them resulted as present in our list of 1, 643 mutated genes (flagged in Suppl File S2) and were excluded from this analysis.

Moreover, mutated genes were catalogued according to a selection of eight pathways chosen on the basis of the existing knowledge about MM and lymphoid tumor malignancies [23, 24]. KEGG database (http://www.genome.jp/kegg/) was used to retrieve full lists of genes composing each pathway (Supplementary Methods).

### Gene expression profiles of mutated genes

A panel of highly purified bone marrow PCs from 55 MM and 21 pPCL patients (12 of whom investigated in this study) and from four healthy donors were previously profiled by us on GeneChip Human Gene 1.0 ST array (Affymetrix, Santa Clara, CA, USA) [12]. These data were used to investigate gene expression levels of mutated genes and to perform hierarchical clustering and differential expression analysis (Supplementary Methods). To assess the clustering abilities of mutated genes, the biological homogeneity index (BHI) [54] provided in R package *clValid* was used (Supplementary Methods).

## SUPPLEMENTARY DATA TABLES AND FIGURES












