# Supplementary material for: Whole-exome sequencing of primary plasma cell leukemia discloses heterogeneous mutational patterns
Source: Oncotarget. 2015 May 25;6(19):17543–58. doi: 10.18632/oncotarget.4028 (PMC4627327; doi:10.18632/oncotarget.4028)
Supplement: Supplementary file 1 [file oncotarget-06-17543-s001.pdf]

SUPPLEMENTARY DATA

WHOLE-EXOME CAPTURE AND SEQUENCING (WES)

Total genomic DNA samples were extracted using Wizard Genomic DNA Purification kit (Promega, Milan, Italy), quantified by ND-1000 spectrophotometer (NanoDrop Technologies, Wilmington, DE, USA) and stored at –20°C until use. Starting from 1 ug of genomic DNA per sample, library preparation and whole-exome capture for all the 12 PCL and five control samples were performed using the Illumina TruSeq DNA Sample Preparation kit and TruSeq Exome Enrichment kit (Illumina, San Diego, CA, USA), according to manufacturer's instructions. The Illumina TruSeq Exome Enrichment system is an in-liquido capture method based on biotinylated bait probes purposely designed to capture and isolate all the coding sequences of the human genome as well as a portion of flanking regions, for a total of 62 Mb. Briefly, 1 ug of genomic DNA per sample was sheared using a Covaris S2 AFA instrument (Covaris, Woburn, MA, USA) and checked for fragmentation by microcapillary electrophoresis on Agilent 2100 BioAnalyzer (Agilent Technologies, Santa Clara, CA, USA). Then, genomic DNA library was prepared according to standard Illumina protocol, including end-repair, A-tailing, adapter ligation and PCR amplification. Next, whole-exome enrichment was carried out by two 20 hour-hybridizations with biotinylated capture baits, each followed by streptavidin magnetic bead binding and washing, and a final post-capture PCR amplification. The enriched libraries were loaded, one sample per lane, onto the Illumina cBot Cluster Generation System at a 8 pM final concentration, and then sequenced by Illumina GAIIx instrument in paired-end 85-cycle runs. For raw data processing, the Sequencing Control Software (SCS, Illumina) was used to convert raw image data into qseq files and then CASAVA software (Illumina) to generate final fastq files.

WES DATA ANALYSIS

Variant calling

After fastq file quality control by using FastQC tool (http://www.bioinformatics.babraham.ac.uk/projects/fastqc/), we implemented a custom bioinformatics pipeline based on open-source tools for automation of read mapping and variant calling and annotation. Basically, whole-exome sequencing (WES) paired-end reads were mapped to the NCBI human reference genome GRCh37 using the Burrows-Wheeler Aligner [1] (BWA, v.0.7.5, http://bio-bwa.sourceforge.net/) with default parameters. The human reference genome was obtained from the GATK ftp site (http://ftp.broadinstitute.org) to have a reference sequence already formatted for the correct execution of GATK. The data bundle v.2.5 was downloaded, including the human reference genome (b37 format, Standard 1000 Genomes fasta) with supercontigs (which were not considered for our analysis). After duplicate removal by Samtools (http://samtools.sourceforge.net/), GATK software [2] (v.2.5) was used to perform quality score recalibration (using the *TableRecalibration* walker), local realignment around known indels (using the *IndelRealigner* walker) and variant calling (by the *UnifiedGenotyper* walker) for both single nucleotide variants (SNVs) and insertions/deletions (indels) on the tumor samples. Poorly confident variants having QUAL < 150, Fisher Strand (FS) strand bias > 60 for SNV and > 200 for indels, or three SNVs within 10 base-windows were flagged for removal in the FILTER field of the VCF file.

Variant annotation

Finally, functional annotation and impact prediction were performed according to the Ensembl GenCode v67 transcript database, and using both Annovar tool [3] (v2013Nov17), which includes five damage predictors for non-synonymous SNVs (SIFT, Polyphen2, LRT, MutationTaster and Mutation Assessor), and SIFT Indel [4] for impact prediction of frameshifting indels.

Filtering

Since we were primarily interested in coding regions and splice sites, raw variants were progressively filtered to obtain a sub-selection of coding non-silent variants comprising both non-synonymous SNVs and indels. The following criteria were applied for filtering: (i) variant must be covered by least 10 reads (read depth ≥ 10x); (ii) variant must be mapped in coding regions or splice sites, according to Ensembl v67 functional annotation; (iii) allele frequency (AF) of non-reference alternate allele (ALT) is ≥ 0.3; (iv) variant is not present in NCBI dbSNP137 database (including also Phase I 1000 Genomes Project data) nor in NHLBI Exome Sequencing Project (ESP6500) database, or even if annotated as known polymorphism, it is also included in the Catalogue Of Somatic Mutations In Cancer (COSMIC, v67) or in the NCBI ClinVar database (database for rs with clinical associations); (v) variant is completely absent in our internal non-tumor WES database (to discard sequencing or analysis error-prone positions); (vi) variant is non-silent, namely a non-synonymous SNV or an indel. For indels, cross-check with public and internal databases was performed by comparing indel start positions extended of 5 bp on both sides to take into account possible overlapping calls.

Selection of coding somatic non-syn SNVs and indels

Next, since we were interested into somatic variants, we used control samples to remove germline background and select tumor-specific non-silent variants. The analysis on control samples was restricted only to the genomic positions called as coding non-silent variants in tumor cases (according to the criteria above described). SNV calls were compared on punctual positions, while indel locations were extended of 5 bp on both sides to take into account possible overlapping calls. Basically, the five control samples were first considered and analyzed as a pool, by using the same GATK walkers used for tumor cases. When having few samples, this approach allows to be more confident on low frequency/quality calls, otherwise discarded in single-sample analysis. Then, for the five paired pPCLs having matched control (cases no. 016, 018, 019, 026, 038), variant calls for each sample were extracted from the pooled analysis and used separately for individual tumor-vs-control comparisons; conversely, for the seven unpaired pPCLs (cases no. 017, 020, 027, 030, 032, 035, 036), variant calls of the five controls were used as a unique pool. For the five paired pPCLs, we considered as "somatic", and maintained, those coding non-syn SNVs and indels that: (i) have an ALT AF ≥ 0.3 in tumor sample; (ii) are absent in matched control sample, with at least 5x depth (flagged as SURE in Supplementary File S1); iii) have an ALT AF < 0.2 in matched control sample (with at least 5x depth) but at least doubled their AF in tumor as compared to control sample (ratio AF tumor/AF control ≥ 2) (flagged as CONFIDENT in Supplementary File S1). For the seven unpaired pPCLs, we considered as "somatic", and maintained, those coding non-syn SNVs and indels that: (i) have an AF ≥ 0.3 in tumor sample; (ii) are completely absent in the control pool. As above described, SNVs were compared by punctual positions, while indels were extended of 5 bp on both sides to take into account overlapping calls.

Identification of coding somatic sub-clonal variants

Since PCL is a notoriously heterogeneous disease, we evaluated the presence of somatic sub-clonal lesions in the five paired pPCLs. Since working below the thresholds commonly set for confident variant calling, this kind of analysis can be performed only on paired samples, which give the possibility to detect low frequency variants and to compare their AF in tumor with respect to normal counterpart in order to exclude sequencing errors and identify real sub-clonal events. Starting from the initial raw calls of the five paired pPCLs, we applied all the criteria aforementioned in the "Filtering paragraph", with the only difference here, at step (iii), of keeping variants with ALT AF < 0.3. Thus, we selected coding sub-clonal non-silent variants (sub-clonal non-syn SNVs and indels). Then, we removed germline background using the five matched control samples, which were first analyzed as a pool and then used as separate for individual tumor-vs-control comparisons (as described in the previous paragraph). The analysis on control samples was restricted only to the genomic positions called as coding sub-clonal non-silent variants in tumor cases. We considered as "somatic sub-clonal", and maintained, those non-syn SNVs and indels that: (i) have an ALT AF < 0.3 in tumor sample; (ii) are absent in matched control sample (flagged as SUBCLONAL SURE in Supplementary File S1); iii) have an ALT AF < 0.1 in its matched control (using the calls made starting from control pool) but at least doubled their AF in tumor as compared to control sample (ratio AF tumor/AF control ≥ 2) (flagged as SUBCLONAL CONFIDENT in Supplementary File S1). SNVs were compared by punctual positions, while indels were extended of 5 bp on both sides to take into account possible overlapping calls. In our experience, GATK is able to call variants with AF till to 0.1, in line with what confirmed by two recent papers [5, 6]. This AF range from 0.3 to 0.1 was our window for sub-clonal variant calling.

COPY NUMBER ANALYSIS FROM WES DATA

Genome-wide analysis of somatic DNA copy number alterations (CNA) was performed starting from WES data using the read count-based EXCAVATOR software [7], with default parameters and setting $c = 1$. The five paired pPCLs (cases no. 016, 018, 019, 026, 038) were analyzed according to the "somatic" analysis mode by comparing each pPCL to its matched control sample, while the seven unpaired pPCLs (cases no. 017, 020, 027, 030, 032, 035, 036) were analyzed with the "pooling" modality by comparing each tumor to the pool of the five control samples. After a careful evaluation of the size of somatic CNAs resulting from the paired analyses, we decided to exclude any alterations less than 200 Kb in the unpaired analyses to filter out likely residual germline CNVs and focus on somatic CNAs. The Circos plot was used to summarize and represent CNA regions found in each pPCL according to a five copy number (CN) state code (ranging from 2-copy loss to 3-copy gain).

PATHWAY ANALYSIS FOR MUTATED GENES

After removal of potentially spurious genes frequently mutated in cancer but not necessarily relevant to tumor biology [8], the mutated gene list (n = 1, 620) was submitted to ToppGene Suite portal (http://toppgene

.cchmc.org) for functional enrichment analysis by using ToppFun application [9]. A *p*-value cut-off of 0.05 and FDR Benjamini-Hochberg (FDR B&H) correction method were applied to all the annotation terms in the default parameter set, and pathways with *q*-value < 0.05 (FDR B&H correction) were defined as significantly enriched.

In addition, mutated genes identified in our pPCL dataset were catalogued according to a selection of eight biological pathways chosen on the basis of the already existing knowledge about MM and lymphoid tumor malignancies [10, 11]. The Kyoto Encyclopedia of Genes and Genomes (KEGG) database (http://www.genome.jp/kegg/) was used to retrieve the full lists of genes composing each pathway: proteasome (map#03050, 48 genes), spliceosome (map#03040, 120 genes), ubiquitin-mediated proteolysis (map#04120, 119 genes), TNF pathway (map#04688, 82 genes), NF-kB pathway (map#04064, 80 genes), PI3K-Akt pathway (map#04151, 221 genes), MAPK pathway (map#04010, 181 genes), Hippo pathway (map#04390, 83 genes). Genes were screened in our pPCLs using both official Gene Name and aliases, and assigned to a given pathway if presenting at least one coding somatic non-syn SNV or indel in at least one case.

GENE EXPRESSION PROFILES OF MUTATED GENES IN 55 MM AND 21 PPCL DATASET

A panel of highly purified bone marrow plasma cells from 55 MM and 21 pPCL patients (12 of whom investigated in this study) and from four healthy donors were previously profiled by us on GeneChip Human Gene 1.0 ST array (Affymetrix, Santa Clara, CA, USA) [12]. MM samples were stratified in five groups according to the traditional cytogenetic classification [13, 14], as already reported [12]. Raw intensity expression values were processed by Robust Multi-array Average (RMA) procedure, using the re-annotated Chip Definition File from BrainArray libraries (version 18.0.0, available at http://brainarray.mbni.med.umich.edu). The most variable genes (2AVEFC) across the whole dataset were defined as those showing an average change in expression level at least 2-fold from the mean across the considered dataset, as previously reported [15]. Hierarchical agglomerative clustering was carried out using Pearson correlation and average as distance and linkage methods, respectively, in dChip software. Two-class supervised analysis was carried out using the Significance Analysis of Microarrays (SAM) software (version 4.00, Excel front-end publicly available at http://www-stat.stanford.edu/Btibs/SAM/index.html). The cut-off for statistical significance (*q*-value = 0) was determined by tuning the delta parameter on the false discovery rate and controlling the *q*-value of the selected probes.

To assess the clustering abilities of the 132 most variable and mutated genes identified by our analysis, the biological homogeneity index (BHI) [16] provided in R package *clValid* was used. The expression values of these 132 genes were used to perform hierarchical clustering of the 55 MM-21 pPCL dataset obtaining seven sample groups, while tumor type and the classes of translocations were set as categories according to which homogeneity was measured using BHI index. Statistical significance was tested calculating BHI index on 1, 000 partitions into seven subgroups of the initial dataset obtained by random sampling of other 132 2AVEFC genes, not including the initial set of 132 most variable and mutated genes.

REFERENCES

1. Li H, Durbin R. Fast and accurate short read alignment with Burrows-Wheeler transform. Bioinformatics. 2009; 25:1754–60.
2. DePristo MA, Banks E, Poplin R, Garimella KV, Maguire JR, Hartl C, Philippakis AA, del Angel G, Rivas MA, Hanna M, McKenna A, Fennell TJ, Kernytsky AM, et al. A framework for variation discovery and genotyping using next-generation DNA sequencing data. Nat Genet. 2011; 43:491–498.
3. Wang K, Li M, Hakonarson H. ANNOVAR: functional annotation of genetic variants from high-throughput sequencing data. Nucleic acids research. 2010; 38:e164.
4. Hu J, Ng PC. Predicting the effects of frameshifting indels. Genome biology. 2012; 13:R9.
5. Xu H, DiCarlo J, Satya RV, Peng Q, Wang Y. Comparison of somatic mutation calling methods in amplicon and whole exome sequence data. BMC genomics. 2014; 15:244.
6. Spencer DH, Tyagi M, Vallania F, Bredemeyer AJ, Pfeifer JD, Mitra RD, Duncavage EJ. Performance of common analysis methods for detecting low-frequency single nucleotide variants in targeted next-generation sequence data. The Journal of molecular diagnostics: JMD. 2014; 16:75–88.
7. Magi A, Tattini L, Cifola I, D'Aurizio R, Benelli M, Mangano E, Battaglia C, Bonora E, Kurg A, Seri M, Magini P, Giusti B, Romeo G, et al. EXCAVATOR: detecting copy number variants from whole-exome sequencing data. Genome Biol. 2013; 14:R120.
8. Lawrence MS, Stojanov P, Polak P, Kryukov GV, Cibulskis K, Sivachenko A, Carter SL, Stewart C, Mermel CH, Roberts SA, Kiezun A, Hammerman PS, McKenna A, et al. Mutational heterogeneity in cancer and

the search for new cancer-associated genes. Nature. 2013; 499:214–8.

9. Chen J, Bardes EE, Aronow BJ, Jegga AG. ToppGene Suite for gene list enrichment analysis and candidate gene prioritization. Nucleic acids research. 2009; 37:W305–11.
10. Abdi J, Chen G, Chang H. Drug resistance in multiple myeloma: latest findings and new concepts on molecular mechanisms. Oncotarget. 2013; 4:2186–207.
11. Bommert K, Bargou RC, Stühmer T. Signalling and survival pathways in multiple myeloma. European journal of cancer. 2006; 42:1574–80.
12. Todoerti K, Agnelli L, Fabris S, Lionetti M, Tuana G, Mosca L, Lombardi L, Grieco V, Bianchino G, D'Auria F, Statuto T, Mazzoccoli C, De Luca L, et al. Transcriptional characterization of a prospective series of primary plasma cell leukemia revealed signatures associated with tumor progression and poorer outcome. Clin Cancer Res. 2013; 19:3247–58.
13. Agnelli L, Bicciato S, Mattioli M, Fabris S, Intini D, Verdelli D, Baldini L, Morabito F, Callea V, Lombardi L, Neri A. Molecular classification of multiple myeloma: a distinct transcriptional profile characterizes patients expressing CCND1 and negative for 14q32 translocations. Journal of clinical oncology. 2005; 23:7296–306.
14. Hideshima T, Bergsagel PL, Kuehl WM, Anderson KC. Advances in biology of multiple myeloma: clinical applications. Blood. 2004; 104:607–18.
15. Mattioli M, Agnelli L, Fabris S, Baldini L, Morabito F, Bicciato S, Verdelli D, Intini D, Nobili L, Cro L, Pruneri G, Callea V, Stelitano C, et al. Gene expression profiling of plasma cell dyscrasias reveals molecular patterns associated with distinct IGH translocations in multiple myeloma. Oncogene. 2005; 24:2461–73.
16. Datta S, Datta S. Methods for evaluating clustering algorithms for gene expression data using a reference set of functional classes. BMC bioinformatics. 2006; 7:397.

Supplementary Table S1. Whole-exome sequencing statistics.

Sample	No. raw reads	No. uniquely mapped reads	No. mapped reads after dup removal	Mean read depth on target	Target coverage	Target coverage (at 10x)
PCL-016	110,272,584	106,445,251 (96.5%)	89,596,142 (81.2%)	50.4x	97.1%	91.5%
PCL-017	84,2,58,526	83,154,938 (98.6%)	69,269,562 (82.2%)	35.3x	96.7%	87.4%
PCL-018	92,253,590	90,678,466 (98.2%)	82,754,489 (89.7%)	40.8x	97.1%	89.3%
PCL-019	78,589,656	77,245,533 (98.2%)	70,705,473 (89.9%)	37.6x	96.9%	87.5%
PCL-020	122,929,488	121,165,743 (98.5%)	112,755,961 (91.7%)	50.6x	97.7%	91.3%
PCL-026	76,134,384	74,597,368 (97.9%)	67,990,361 (89.3%)	32.5x	96.8%	87.1%
PCL-027	98,189,514	97,011,769 (98.8%)	79,048,609 (80.5%)	42.5x	97.0%	90.1%
PCL-030	95,130,580	93,779,211 (98.5%)	84,904,631 (89.2%)	42.6x	96.7%	89.2%
PCL-032	96,285,158	92,118,072 (95.6%)	76,863,539 (79.8%)	39.9x	96.8%	88.2%
PCL-035	90,835,438	89,744,765 (98.7%)	82,849,366 (91.2%)	42.6x	96.8%	90.1%
PCL-036	88,514,304	87,475,902 (98.8%)	77,571,653 (87.6%)	40.4x	97.0%	89.0%
PCL-038	108,860,542	107,692,450 (98.9%)	98,740,986 (90.7%)	50.5x	97.1%	91.4%
PCL-016 CTRL	105,707,714	104,019,558 (98.4%)	91,154,951 (86.2%)	47.8x	97.2%	92.1%
PCL-018 CTRL	80,980,836	79,894,617 (98.6%)	66,856,222 (82.5%)	35.9x	96.6%	87.7%
PCL-019 CTRL	85,963,624	84,531,674 (98.3%)	70,913,619 (82.4%)	39.0x	97.0%	89.8%
PCL-026 CTRL	82,031,414	80,110,921 (97.6%)	72,186,348 (87.9%)	38.1x	97.0%	89.9%
PCL-038 CTRL	123,518,132	121,662,698 (98.4%)	103,777,057 (84.0%)	52.1x	97.4%	92.4%

Supplementary Table S2. Single nucleotide variants (SNVs) and insertion/deletion (INDELs) counts by sample.

Sample	SNVs					INDELs			
	Raw calls	Passed 10x	Coding	Novel Coding	Novel Coding Non-syn	Raw calls	Passed 10x	Coding	Novel Coding
PCL-016	49330	42882	18713	3277	1755	5052	4638	256	106
PCL-017	47048	37830	16373	2705	1409	4435	3907	272	130
PCL-018	48100	39959	17244	2834	1463	4652	4177	274	133
PCL-019	46853	38512	16832	2938	1588	4575	4024	275	139
PCL-020	47983	41292	18085	3007	1565	4986	4573	288	144
PCL-026	47489	37774	16367	2933	1579	4434	3882	262	123
PCL-027	48185	40804	17662	2825	1461	4898	4401	295	136
PCL-030	48009	40542	17609	2854	1499	4900	4415	282	124
PCL-032	46979	39390	17149	2795	1445	4628	4131	263	113
PCL-035	48580	41497	17997	3010	1548	4946	4524	289	134
PCL-036	47383	39829	17207	2774	1435	4856	4402	297	139
PCL-038	47370	40591	17633	2951	1552	4777	4369	286	139

Supplementary Table S3. Selected pathways found affected in our pPCL series and known to be involved in plasma cell dyscrasias.

Pathway	No. Affected genes (%)	No. pPCL (%)	No. Damaging/ Total variants	Genes
Proteasome	1 (2%)	1 (8%)	1/1	PSMC5
Spliceosome	9 (7.5%)	9 (75%)	5/11	ACIN1, CRNKL1, CTNNBL1, DDX42, HSPA8, NCBP1, SNRNP200, WBP11, XAB2
Ubiquitin-mediated proteolysis	9 (7.5%)	5 (42%)	6/10	BIRC2*, BRCA1, DDB2*, TRAF6, TRIM37, TRIP12, UBE3A, UBE4A, UBR5*
TNF pathway	8 (9.7%)	6 (50%)	5/8	CASP7*, CASP8, JUN, MAP3K5, NOD2, TNFRSF1A, TRAF2, TRAF3*
NF-kB pathway	8 (10%)	7 (58%)	5/11	ATM, BIRC2*, PIDD, TNFRSF11A, TNFRSF1A, TRAF2, TRAF3*, TRAF6
PI3K-Akt pathway	25 (11.3%)	12 (100%)	19/32	BRCA1, CDKN1A, FN1, GHR, HGF*, HRAS, IL3RA, ITGA1, JAK1, KIT, KRAS, LAMA2, LAMA3, LAMA4, LAMA5, LAMB1, NRAS, PDGFB, PDGFRA, PDGFRB, PRLR, RELN, TLR2*, TP53, TSC2
MAPK pathway	22 (12.1%)	12 (100%)	21/27[†]	BDNF, BRAF, CACNA2D1, CACNA2D3, HRAS, HSPA8, JUN, KRAS, MAP3K5, MAPK8IP3, MAX*, MEF2C, MST1, NRAS, PDGFB, PDGFRA, PDGFRB, TGFBR2, TNFRSF1A, TP53, TRAF2, TRAF6
Hippo pathway	4 (4.8%)	3 (25%)	4/4	AREG, FZD10, FZD6, TGFBR2

The percentage of affected genes per pathway was calculated on the total number of genes included in each process, as defined from KEGG database.

[*] Truncating variants (nonsense mutations or frameshift indels).

[†] Significant enrichment for damaging variants (q-value < 0.01, Bonferroni correction).

Supplementary Table S4. Coding somatic variants in the panel of 11 genes recurrently mutated in multiple myeloma.

Gene name	no. affected PCLs	no. variants	Non-syn SNVs and indels by sample	CN alterations across the 12 PCLs	Same variant as previously reported in multiple myeloma
ACTG1	-	-	-	-	
BRAF	1	2	PCL-026 (D594N†, E586K†)	-	Boyd *et al.*, BJH 2011 (D594N); Bolli *et al.*, Nat Commun 2014 (E586K)
CYLD	-	-	-	-	
DIS3	3	2	PCL-019 (R780K†), PCL-035 (R780T), PCL-036 (H764RΔ)	1-copy loss in 8 PCLs (016, 017, 018, 026, 030, 032, 036, 038)	Walker *et al.*, Blood 2012 (R780K); Lohr *et al.*, Cancer Cell 2014 (R780T)
FAM46C	1	1	PCL-027 (S156F)	1-copy loss in 5 PCLs (017, 019, 032, 036, 038)	
KRAS	1	1	PCL-038 (G12R†)	1-copy loss in PCL-030	Walker *et al.*, Blood 2012 (G12R)
NRAS	1	1	PCL-027 (Q61H†)	1-copy loss in 5 PCLs (017, 019, 032, 036, 038), 1-copy gain in PCL-027	Chapman *et al.*, Nature 2011 (Q61H)
PRDM1	-	-	-	-	
RB1	-	-	-	-	
TP53	4	4	PCL-018 (P278LΔ†), PCL-027 (I195TΔ†), PCL-030 (R273CΔ†), PCL-017 (332fs*delΔ)	1-copy loss in 5 PCLs (017, 018, 019, 027, 030)	
TRAF3	1	1	PCL-032 (C21fs*delΔ)	2-copy loss in PCL-017, 1-copy loss in PCL-032	

CN state was evaluated only for those genes affected by at least one coding somatic non-silent variant (non-syn SNV or indel) in our dataset.

Legend: Δ variants in homozygous state † variants reported as somatic in COSMIC (v67) for other tumors.

Supplementary Table S5. List of the 132 most variable and mutated genes. Functional enrichment in Gene Ontology (GO) functional annotation terms is reported as calculated by DAVID v6.7 software (Enrichment Score > 1.3).

Gene symbol	Gene name	GO functional enrichment
ABCB4	ATP-binding cassette, sub-family B (MDR/TAP), member 4	GOTERM_CC: integral to plasma membrane (GO:0005887)
ADAMTS15	ADAM metallopeptidase with thrombospondin type 1 motif, 15	GOTERM_CC: proteinaceous extracellular matrix (GO:0005578)
ADRB2	adrenergic, beta-2-, receptor, surface	GOTERM_BP: positive regulation of developmental process (GO:0051094); GOTERM_BP: endocytosis (GO:0006897); GOTERM_CC: integral to plasma membrane (GO:0005887)
AMPD1	adenosine monophosphate deaminase 1 (isoform M)	
ANG	angiogenin, ribonuclease, RNase A family, 5	GOTERM_CC: proteinaceous extracellular matrix (GO:0005578); GOTERM_MF: cytoskeletal protein binding (GO:0008092)
APH1B	anterior pharynx defective 1 homolog B (C. elegans)	
ARHGAP15	Rho GTPase activating protein 15	
ARHGAP31	Cdc42 GTPase-activating protein	
ASPM	asp (abnormal spindle) homolog, microcephaly associated (Drosophila)	
ATRNL1	attractin-like 1	
BAI3	brain-specific angiogenesis inhibitor 3	
BTLA	B and T lymphocyte associated	GOTERM_CC: integral to plasma membrane (GO:0005887)
C17orf47	chromosome 17 open reading frame 47	
CABLES1	Cdk5 and Abl enzyme substrate 1	
CARD6	caspase recruitment domain family, member 6	
CCSER1	coiled-coil serine-rich protein 1	
CD36	CD36 molecule (thrombospondin receptor)	GOTERM_BP: positive regulation of developmental process (GO:0051094); GOTERM_BP: cell adhesion (GO:0007155); GOTERM_BP: endocytosis (GO:0006897); GOTERM_CC: integral to plasma membrane (GO:0005887)
CFI	complement factor I	
CHSY3	chondroitin sulfate synthase 3	
CLSTN2	calsyntenin 2	GOTERM_BP: cell adhesion (GO:0007155)
COBLL1	COBL-like 1	

(*Continued*)

Gene symbol	Gene name	GO functional enrichment
COL1A2	collagen, type I, alpha 2	GOTERM_BP: extracellular structure organization (GO:0043062); GOTERM_CC: proteinaceous extracellular matrix (GO:0005578)
CPNE8	copine VIII	
CRYZ	crystallin, zeta (quinone reductase)	
CYBB	cytochrome b-245, beta polypeptide	GOTERM_CC: integral to plasma membrane (GO:0005887)
DAB2	disabled homolog 2, mitogen-responsive phosphoprotein (Drosophila)	GOTERM_BP: cell morphogenesis (GO:0000902); GOTERM_BP: endocytosis (GO:0006897)
DAPK1	death-associated protein kinase 1	
DDX60	DEAD (Asp-Glu-Ala-Asp) box polypeptide 60	
DLGAP5	discs, large (Drosophila) homolog-associated protein 5	GOTERM_MF: phosphoprotein phosphatase activity (GO:0004721)
DOCK4	dedicator of cytokinesis 4	
DUSP4	dual specificity phosphatase 4	GOTERM_MF: phosphoprotein phosphatase activity (GO:0004721)
EML5	echinoderm microtubule associated protein like 5	
ETV1	ets variant 1	
FAM129A	family with sequence similarity 129, member A	
FAM171A1	family with sequence similarity 171, member A1	
FAM5C	family with sequence similarity 5, member C	
FAT4	FAT tumor suppressor homolog 4 (Drosophila)	GOTERM_BP: cell adhesion (GO:0007155)
FLNB	filamin B, beta (actin binding protein 278)	GOTERM_MF: cytoskeletal protein binding (GO:0008092)
FN1	fibronectin 1	GOTERM_BP: cell morphogenesis (GO:0000902); GOTERM_BP: cell adhesion (GO:0007155); GOTERM_CC: proteinaceous extracellular matrix (GO:0005578)
FNBP1	formin binding protein 1	GOTERM_BP: endocytosis (GO:0006897)
GABRG1	gamma-aminobutyric acid (GABA) A receptor, gamma 1	
GBA3	glucosidase, beta, acid 3 (cytosolic)	
GBP4	guanylate binding protein 4	
GLUL	glutamate-ammonia ligase (glutamine synthetase)	
GRAMD3	GRAM domain containing 3	
GREB1	GREB1 protein	
HGF	hepatocyte growth factor (hepapoietin A; scatter factor)	GOTERM_BP: cell morphogenesis (GO:0000902)

(Continued)

Gene symbol	Gene name	GO functional enrichment
HIST1H2BK	histone cluster 1, H2bk	
HSPA4L	heat shock 70kDa protein 4-like	
KCNA5	potassium voltage-gated channel, shaker-related subfamily, member 5	GOTERM_CC: integral to plasma membrane (GO:0005887)
KIAA1598	KIAA1598	
KIT	v-kit Hardy-Zuckerman 4 feline sarcoma viral oncogene homolog	GOTERM_BP: positive regulation of developmental process (GO:0051094)
KMO	kynurenine 3-monooxygenase (kynurenine 3-hydroxylase)	
LAMA2	laminin, alpha 2	GOTERM_BP: cell adhesion (GO:0007155); GOTERM_CC: proteinaceous extracellular matrix (GO:0005578)
LEPREL1	leprecan-like 1	
LGALS3BP	lectin, galactoside-binding, soluble, 3 binding protein	GOTERM_BP: cell adhesion (GO:0007155); GOTERM_CC: proteinaceous extracellular matrix (GO:0005578)
LIFR	leukemia inhibitory factor receptor alpha	GOTERM_BP: cell morphogenesis (GO:0000902); GOTERM_CC: integral to plasma membrane (GO:0005887)
LPHN3	latrophilin 3	
LRMP	lymphoid-restricted membrane protein	GOTERM_CC: integral to plasma membrane (GO:0005887)
LRP12	low density lipoprotein-related protein 12	GOTERM_BP: endocytosis (GO:0006897); GOTERM_CC: integral to plasma membrane (GO:0005887)
MAGEC1	melanoma antigen family C, 1	
MAGI2	membrane associated guanylate kinase, WW and PDZ domain containing 2	
MAP1B	microtubule-associated protein 1B	GOTERM_BP: positive regulation of developmental process (GO:0051094); GOTERM_BP: cell morphogenesis (GO:0000902); GOTERM_BP: extracellular structure organization (GO:0043062); GOTERM_MF: cytoskeletal protein binding (GO:0008092)
MBOAT1	membrane bound O-acyltransferase domain containing 1	
MLIP	muscular LMNA-interacting protein	
MSC	musculin (activated B-cell factor-1)	
MUC15	mucin 15, cell surface associated	
MYBL1	v-myb myeloblastosis viral oncogene homolog (avian)-like 1	

(*Continued*)

Gene symbol	Gene name	GO functional enrichment
MYO1B	myosin IB	GOTERM_MF: cytoskeletal protein binding (GO:0008092)
NAP1L3	nucleosome assembly protein 1-like 3	
NEB	nebulin	GOTERM_MF: cytoskeletal protein binding (GO:0008092)
NLGN4X	neuroligin 4, X-linked	GOTERM_BP: cell adhesion (GO:0007155); GOTERM_CC: integral to plasma membrane (GO:0005887); GOTERM_BP: extracellular structure organization (GO:0043062)
NRCAM	neuronal cell adhesion molecule	GOTERM_BP: positive regulation of developmental process (GO:0051094); GOTERM_BP: cell morphogenesis (GO:0000902); GOTERM_BP: cell adhesion (GO:0007155); GOTERM_CC: integral to plasma membrane (GO:0005887); GOTERM_BP: extracellular structure organization (GO:0043062); GOTERM_MF: cytoskeletal protein binding (GO:0008092)
NTRK3	neurotrophic tyrosine kinase, receptor, type 3	GOTERM_BP: positive regulation of developmental process (GO:0051094); GOTERM_CC: integral to plasma membrane (GO:0005887)
NXPE1	neurexophilin and PC-esterase domain family, member 1	
P4HA1	prolyl 4-hydroxylase, alpha polypeptide I	GOTERM_BP: extracellular structure organization (GO:0043062)
PCDH20	protocadherin 20	GOTERM_BP: cell adhesion (GO:0007155)
PDIA2	protein disulfide isomerase family A, member 2	
PDLIM3	PDZ and LIM domain 3	GOTERM_MF: cytoskeletal protein binding (GO:0008092)
PER1	period homolog 1 (Drosophila)	
PLBD1	phospholipase B domain containing 1	
PLCB1	phospholipase C, beta 1 (phosphoinositide-specific)	
PLCL1	phospholipase C-like 1	
PNP	nucleoside phosphorylase	GOTERM_BP: positive regulation of developmental process (GO:0051094)
PRDM5	PR domain containing 5	
PTCH1	patched homolog 1 (Drosophila)	GOTERM_CC: integral to plasma membrane (GO:0005887)
PTGDR	prostaglandin D2 receptor (DP)	
PTPN13	protein tyrosine phosphatase, non-receptor type 13 (APO-1/CD95 (Fas)-associated phosphatase)	GOTERM_MF: phosphoprotein phosphatase activity (GO:0004721)

(*Continued*)

Gene symbol	Gene name	GO functional enrichment
PTPRC	protein tyrosine phosphatase, receptor type, C	GOTERM_BP: positive regulation of developmental process (GO:0051094); GOTERM_MF: phosphoprotein phosphatase activity (GO:0004721); GOTERM_BP: cell adhesion (GO:0007155); GOTERM_CC: integral to plasma membrane (GO:0005887)
PTPRG	protein tyrosine phosphatase, receptor type, G	GOTERM_MF: phosphoprotein phosphatase activity (GO:0004721); GOTERM_CC: integral to plasma membrane (GO:0005887)
PTPRZ1	protein tyrosine phosphatase, receptor-type, Z polypeptide 1	GOTERM_BP: cell morphogenesis (GO:0000902); GOTERM_MF: phosphoprotein phosphatase activity (GO:0004721); GOTERM_CC: integral to plasma membrane (GO:0005887); GOTERM_CC: proteinaceous extracellular matrix (GO:0005578)
PXDN	peroxidasin homolog (Drosophila)	
QPCT	glutaminyl-peptide cyclotransferase	
RAPGEF4	Rap guanine nucleotide exchange factor (GEF) 4	
RELN	reelin	GOTERM_BP: cell morphogenesis (GO:0000902); GOTERM_BP: cell adhesion (GO:0007155); GOTERM_CC: proteinaceous extracellular matrix (GO:0005578)
ROBO1	roundabout, axon guidance receptor, homolog 1 (Drosophila); similar to roundabout 1 isoform b	GOTERM_BP: positive regulation of developmental process (GO:0051094); GOTERM_BP: cell morphogenesis (GO:0000902); GOTERM_BP: cell adhesion (GO:0007155); GOTERM_CC: integral to plasma membrane (GO:0005887)
SAMD3	sterile alpha motif domain containing 3	
SCN9A	sodium channel, voltage-gated, type IX, alpha subunit	GOTERM_CC: integral to plasma membrane (GO:0005887)
SEMA3A	sema domain, immunoglobulin domain (Ig), short basic domain, secreted, (semaphorin) 3A	GOTERM_BP: cell morphogenesis (GO:0000902)
SEMA4A	sema domain, immunoglobulin domain (Ig), transmembrane domain (TM) and short cytoplasmic domain, (semaphorin) 4A	
SEMA7A	semaphorin 7A, GPI membrane anchor (John Milton Hagen blood group)	
SEPT10	septin 10	
SLA	Src-like-adaptor	
SLAMF1	signaling lymphocytic activation molecule family member 1	
SLC15A2	solute carrier family 15 (H+/peptide transporter), member 2	GOTERM_CC: integral to plasma membrane (GO:0005887)

(*Continued*)

Gene symbol	Gene name	GO functional enrichment
SLC25A27	solute carrier family 25, member 27	-
SLC40A1	solute carrier family 40 (iron-regulated transporter), member 1	GOTERM_CC: integral to plasma membrane (GO:0005887)
SLC9A9	solute carrier family 9 (sodium/hydrogen exchanger), member 9	
SLFN11	schlafen family member 11	
SLIT2	slit homolog 2 (Drosophila)	GOTERM_BP: positive regulation of developmental process (GO:0051094); GOTERM_BP: cell morphogenesis (GO:0000902)
SNX9	sorting nexin 9	
SPG20	spastic paraplegia 20 (Troyer syndrome)	
SPOCK2	sparc/osteonectin, cwcv and kazal-like domains proteoglycan (testican) 2	GOTERM_BP: extracellular structure organization (GO:0043062); GOTERM_CC: proteinaceous extracellular matrix (GO:0005578)
ST14	suppression of tumorigenicity 14 (colon carcinoma)	GOTERM_CC: integral to plasma membrane (GO:0005887)
SYNM	synemin, intermediate filament protein	GOTERM_MF: cytoskeletal protein binding (GO:0008092)
TEX101	testis expressed 101	
TGFBR2	transforming growth factor, beta receptor II (70/80kDa)	GOTERM_BP: positive regulation of developmental process (GO:0051094); GOTERM_BP: endocytosis (GO:0006897); GOTERM_CC: integral to plasma membrane (GO:0005887)
TPTE*	transmembrane phosphatase with tensin homology	GOTERM_MF: phosphoprotein phosphatase activity (GO:0004721)
TPX2	TPX2, microtubule-associated, homolog (Xenopus laevis)	
TRPM3	transient receptor potential cation channel, subfamily M, member 3	
TTN*	titin	GOTERM_MF: cytoskeletal protein binding (GO:0008092)
TXLNB	taxilin beta	
UGT2A1	UDP glucuronosyltransferase 2 family, polypeptide A1	
USH2A	Usher syndrome 2A (autosomal recessive, mild)	GOTERM_CC: proteinaceous extracellular matrix (GO:0005578)
USP6NL	USP6 N-terminal like	
VWDE	von Willebrand factor D and EGF domains	
WDR17	WD repeat domain 17	

(*Continued*)

Gene symbol	Gene name	GO functional enrichment
ZNF154	zinc finger protein 154	
ZNF165	zinc finger protein 165	
ZNF559	zinc finger protein 559	
ZNF83	zinc finger protein 83	
ZNF860	zinc finger protein 860	

Note: * according to Lawrence et al. (Nature 2013), these could be potentially spurious genes frequently found mutated in cancer but not necessarily relevant to tumor biology.

Supplementary Table S6. List of the 102 mutated genes found as differentially expressed (DEGs) in 21 pPCL versus 55 MM comparison, by SAM supervised analysis. Significant DEGs (*q*-value = 0) are listed according to SAM score. Expression fold change in PCL vs MM group is reported. Up- and down-regulated genes belonging to the functionally enriched transcription regulation (T) and apoptosis (A) biological processes, respectively, are indicated.

Gene symbol	Gene name	Cytoband	Score(d)	Fold change	Functional enrichment
USP36	ubiquitin specific peptidase 36	17q25.3	5.67	1.60	-
LGALS3	lectin, galactoside-binding, soluble, 3	14q21-q22	5.03	2.15	-
NFKBIE	nuclear factor of kappa light polypeptide gene enhancer in B-cells inhibitor, epsilon	6p21.1	4.72	2.06	-
PER1	period homolog 1 (Drosophila)	17p13.1-p12	4.25	2.57	T
FAM53B	family with sequence similarity 53, member B	10q26.13	4.16	1.98	-
LSS	lanosterol synthase (2,3-oxidosqualene-lanosterol cyclase)	21q22.3	4.07	1.52	-
ZMIZ2	zinc finger, MIZ-type containing 2	7p13	3.92	1.50	T
WDR44	WD repeat domain 44	Xq24	3.86	1.63	-
TOX2	TOX high mobility group box family member 2	20q13.12	3.81	1.76	T
HIVEP1	human immunodeficiency virus type I enhancer binding protein 1	6p24-p22.3	3.64	1.65	T
MFSD10	major facilitator superfamily domain containing 10	4p16.3	3.61	1.43	-
ESYT1	family with sequence similarity 62 (C2 domain containing), member A	12q13.2	3.57	1.60	-
RNF19B	ring finger protein 19B	1p35.1	3.50	1.74	-
MSC	musculin (activated B-cell factor-1)	8q21	3.48	1.93	T
ZNF43	zinc finger protein 43	19p13.1-p12	3.47	1.71	T
WBP11	WW domain binding protein 11	12p12.3	3.38	1.67	-

(*Continued*)

Gene symbol	Gene name	Cytoband	Score(d)	Fold change	Functional enrichment
FAM129A	family with sequence similarity 129, member A	1q25	3.35	2.24	-
PPIF	peptidylprolyl isomerase F	10q22-q23	3.31	1.51	-
HGS	hepatocyte growth factor-regulated tyrosine kinase substrate	17q25	3.28	1.31	-
SLC20A1	solute carrier family 20 (phosphate transporter), member 1	2q11-q14	3.28	1.57	-
MAPK8IP3	mitogen-activated protein kinase 8 interacting protein 3	16p13.3	3.25	1.38	-
ZNF777	zinc finger protein 777	7q36.1	3.25	1.34	T
ADNP2	ADNP homeobox 2	18q23	3.11	1.59	T
VAV2	vav 2 guanine nucleotide exchange factor	9q34.1	3.06	1.56	-
FAM179A	family with sequence similarity 179, member A	2p23.2	3.06	1.30	-
FUS	fusion (involved in t(12;16) in malignant liposarcoma)	16p11.2	3.05	1.29	-
RARA	retinoic acid receptor, alpha	17q21	3.05	1.37	T
NDE1	nudE nuclear distribution gene E homolog 1 (A. nidulans)	16p13.11	3.04	1.35	-
PLEC	plectin 1	8q24	3.03	1.46	-
PRDM2	PR domain containing 2, with ZNF domain	1p36.21	2.97	1.50	T
CCNK	cyclin K	14q32	2.96	1.39	T
LRRFIP1	leucine rich repeat (in FLII) interacting protein 1	2q37.3	2.94	1.55	T
COBLL1	COBL-like 1	2q24.3	2.94	1.90	-
ZNF687	zinc finger protein 687	1q21.2	2.93	1.41	T
MAP1B	microtubule-associated protein 1B	5q13	2.92	2.18	-
USP14	ubiquitin specific peptidase 14 (tRNA-guanine transglycosylase)	18p11.32	2.89	1.35	-

(*Continued*)

Gene symbol	Gene name	Cytoband	Score(d)	Fold change	Functional enrichment
UHRF1BP1	UHRF1 binding protein 1	6p21	2.87	1.41	-
CEP112	centrosomal protein 112kDa	17q24.1	2.86	1.39	-
BATF3	basic leucine zipper transcription factor, ATF-like 3	1q32.3	2.84	1.46	T
STX4	syntaxin 4	16p11.2	2.84	1.27	-
CCNY	cyclin Y	10p11.21	2.82	1.24	-
EP300	E1A binding protein p300	22q13.2	2.82	1.34	T
SLC33A1	solute carrier family 33 (acetyl-CoA transporter), member 1	3q25.31	−2.65	0.65	A
TMEM117	transmembrane protein 117	12q12	−2.66	0.74	-
MEF2C	myocyte enhancer factor 2C	5q14	−2.66	0.67	A
IPO11	importin 11	5q12.1	−2.67	0.67	-
CEACAM1	carcinoembryonic antigen-related cell adhesion molecule 1 (biliary glycoprotein)	19q13.2	−2.67	0.61	-
PLBD1	phospholipase B domain containing 1	12p13.1	−2.67	0.54	-
TIMD4	T-cell immunoglobulin and mucin domain containing 4	5q33.3	−2.68	0.65	-
ZFP3	zinc finger protein 3 homolog (mouse)	17p13.2	−2.73	0.62	-
CHEK2	checkpoint kinase 2	22q12.1	−2.73	0.71	A
PLCG2	phospholipase C, gamma 2 (phosphatidylinositol-specific)	16q24.1	−2.74	0.67	A
HIST1H2BC	histone cluster 1, H2bc	6p21.3	−2.75	0.61	-
DISP1	dispatched homolog 1 (Drosophila)	1q41	−2.80	0.70	-
BLZF1	basic leucine zipper nuclear factor 1	1q24	−2.81	0.65	-
HMMR	hyaluronan-mediated motility receptor (RHAMM)	5q33.2-qter	−2.82	0.66	-

(*Continued*)

Gene symbol	Gene name	Cytoband	Score(d)	Fold change	Functional enrichment
ZNF233	zinc finger protein 233	19q13.31	−2.82	0.62	-
TTC14	tetratricopeptide repeat domain 14	3q26.33	−2.83	0.66	-
ZNF302	zinc finger protein 302	19q13.11	−2.83	0.63	-
PLD1	phospholipase D1, phosphatidylcholine-specific	3q26	−2.86	0.70	-
ABCD3	ATP-binding cassette, sub-family D (ALD), member 3	1p22-p21	−2.89	0.64	-
FAM46C	family with sequence similarity 46, member C	1p12	−2.90	0.70	-
RNASEL	ribonuclease L (2’,5’-oligoisoadenylate synthetase-dependent)	1q25	−2.93	0.63	-
MAN2A1	mannosidase, alpha, class 2A, member 1	5q21-q22	−2.99	0.69	-
ASXL2	additional sex combs like 2 (Drosophila)	2p24.1	−3.01	0.63	-
KLHL6	kelch-like 6 (Drosophila)		−3.02	0.60	-
RAPGEF4	Rap guanine nucleotide exchange factor (GEF) 4	2q31-q32	−3.03	0.50	-
FXR2	fragile X mental retardation, autosomal homolog 2	17p13.1	−3.05	0.73	-
DLGAP5	discs, large (Drosophila) homolog-associated protein 5	14q22.3	−3.08	0.55	-
WDR73	WD repeat domain 73	15q25.2	−3.09	0.74	-
TM7SF3	transmembrane 7 superfamily member 3	12q11-q12	−3.11	0.69	-
TCAIM	T cell activation inhibitor, mitochondrial	3p21.31	−3.11	0.61	-
RFESD	Rieske (Fe-S) domain containing	5q15	−3.11	0.65	-
MGAM	maltase-glucoamylase (alpha-glucosidase)	7q34	−3.13	0.59	-
ADAL	adenosine deaminase-like	15q15.3	−3.15	0.61	-
BTN3A3	butyrophilin, subfamily 3, member A3	6p21.3	−3.16	0.57	-

(*Continued*)

Gene symbol	Gene name	Cytoband	Score(d)	Fold change	Functional enrichment
QPCT	glutaminyl-peptide cyclotransferase	2p22.2	−3.17	0.28	-
DNAJC10	DnaJ (Hsp40) homolog, subfamily C, member 10	2q32.1	−3.18	0.67	-
MLEC	malectin	12q24.31	−3.23	0.63	-
SLC15A2	solute carrier family 15 (H+/peptide transporter), member 2	3q13.33	−3.26	0.46	-
ANKRD42	ankyrin repeat domain 42	11q14.1	−3.29	0.67	-
RELN	reelin	7q22	−3.33	0.37	-
ITSN1	intersectin 1 (SH3 domain protein)	21q22.1-q22.2	−3.33	0.57	A
NXPE1	neurexophilin and PC-esterase domain family, member 1	11q23.2	−3.33	0.48	-
HS2ST1	heparan sulfate 2-O-sulfotransferase 1	1p31.1-p22.1	−3.34	0.59	-
CHSY3	chondroitin sulfate synthase 3	5q23.3	−3.34	0.44	-
MIXL1	Mix1 homeobox-like 1 (Xenopus laevis)	1q42.12	−3.42	0.59	-
DLC1	deleted in liver cancer 1	8p22	−3.49	0.53	A
DGKI	diacylglycerol kinase, iota	7q32.3-q33	−3.52	0.58	-
AMPD1	adenosine monophosphate deaminase 1 (isoform M)	1p13	−3.60	0.34	-
ATM	ATM serine/threonine kinase (Ataxia telangiectasia mutated)	11q22-q23	−3.64	0.52	A
CPNE5	copine V	6p21.1	−3.66	0.67	-
FAM111A	family with sequence similarity 111, member A	11q12.1	−3.72	0.59	-
APH1B	anterior pharynx defective 1 homolog B (C. elegans)	15q22.2	−3.73	0.47	A
KIAA0040	KIAA0040	1q24-q25	−3.74	0.62	-
FKTN	fukutin	9q31-q33	−3.98	0.58	-
CD36	CD36 molecule (thrombospondin receptor)	7q11.2	−4.09	0.46	-

(*Continued*)

Gene symbol	Gene name	Cytoband	Score(d)	Fold change	Functional enrichment
DENND5B	DENN/MADD domain containing 5B	12p11.21	−4.48	0.57	-
AKD1	adenylate kinase domain containing 1	6q21	−4.49	0.61	-
KMO	kynurenine 3-monooxygenase (kynurenine 3-hydroxylase)	1q42-q44	−4.54	0.36	-
ZNF860	zinc finger protein 860	3p23	−4.64	0.31	-
SLFN11	schlafen family member 11	17q12	−4.86	0.34	-

Supplementary Table S7. Clinical and molecular data of primary plasma cell leukemia cases included in the study.

Case	Age (yr)	Sex	PP	del(13q)	del(17p)	1q gain	t(11;14)	t(4;14)	t(14;16)	t(14;20)	c-MYC rearrangement
PCL-016	57	F	Gκ	1	0	1	0	0	1	0	0
PCL-017	68	F	Gκ	1	1	1	0	0	1	0	1
PCL-018	59	F	κ	1	1	0	1	0	0	0	0
PCL-019	67	F	Mκ	1	1	1	0	0	1	0	0
PCL-020	79	F	Gλ	0	0	1	0	0	0	0	0
PCL-026	59	M	Gκ	1	0	1	0	0	1	0	1
PCL-027	65	M	λ	0	1	0	1	0	0	0	0
PCL-030	52	F	κ	1	1	0	0	0	0	1	0
PCL-032	65	F	Gκ	1	0	1	0	1	0	0	na
PCL-035	76	F	κ	0	0	0	1	0	0	0	0
PCL-036	71	M	Gκ	1	0	0	1	0	0	0	0
PCL-038	58	M	Gκ	1	0	1	0	0	0	0	na

Legend: F, female; M, male; PP, paraprotein; 0, negative; 1, positive; na, not available.

Supplementary Table S8. Estimation of possible carry-over of germline variants when not having matched controls. The five paired pPCL samples were analyzed in both "paired" (subtracting to each tumor its matched control, standard mode) and "unpaired" manner (subtracting to each tumor the pool of the other not matched controls). Only the calls passing all our filtering steps described in Suppl Methods and having AF ≥ 0.3 in tumor were considered. Count comparison between the two analysis modes was performed on non-synonymous SNVs classified as "SURE" (zero alternate reads in control sample, see Supplementary File S1) and not included subclonal variants.

Sample	Paired analysis	Unpaired analysis
PCL-016	146	147
PCL-018	19	21
PCL-019	105	105
PCL-026	224	225
PCL-038	30	31
Total	524	529

Supplementary Table S9. Identical recurrent variants found in two or more samples and annotated in both dbSNP137 and COSMIC catalogue (v67). Check for somatic status as reported in COSMIC website. In COSMIC website, variants are flagged as somatic if confirmed in paired samples or previously reported in literature as somatic; viceversa, variants have a flag "unknown" if normal tissue was not available or not tested (somatic status not confirmed).

Gene Name	Chr_Start	Samples	no. Samples	Flag	dbSNP137 rsID	COSMIC ID (v67)	Somatic Status in COSMIC website
ACACB	12_109692053	PCL-020*PCL-027	2	Unpaired_SNV	rs17848835	COSM1358599, COSM1358600	unknown
BPIFB6	20_31619500	PCL-030*PCL-017*PCL-035*PCL-032	4	Unpaired_SNV	rs17301126	COSM443647	unknown
C16orf96	16_4625470	PCL-020*PCL-030	2	Unpaired_SNV	rs77379438	COSM435266	unknown
CGN	1_151502427	PCL-030*PCL-032	2	Unpaired_SNV	rs34834099	COSM1560000	unknown
CIDEC	3_9918811	PCL-030*PCL-017*PCL-035	3	Unpaired_SNV	rs61742367	COSM1178476	Confirmed somatic
CLSTN2	3_140178381	PCL-036*PCL-020	2	Unpaired_SNV	rs17348572	COSM149480	unknown
DTX2	7_76111831	PCL-020*PCL-035	2	Unpaired_SNV	rs150857694	COSM150315	unknown
EHD4	15_42235316	PCL-036*PCL-035	2	Unpaired_SNV	rs11549015	COSM147887	Previously reported
EPPK1	8_144942413	PCL-036*PCL-017	2	Unpaired_SNV	rs201641573	COSM1330245	unknown
ERICH1	8_623696	PCL-030*PCL-032	2	Unpaired_SNV	rs61741834	COSM328225	unknown
EVC	4_5731074	PCL-017*PCL-032	2	Unpaired_SNV	rs16837598	COSM1618937	Confirmed somatic
EYS	6_65301787	PCL-030*PCL-017	2	Unpaired_SNV	rs12663622	COSM150116	unknown
FAM166B	9_35562393	PCL-036*PCL-035	2	Unpaired_INDEL	rs142582869	COSM288596	Confirmed somatic, Previously reported
FAM179A	2_29246044	PCL-036*PCL-020*PCL-027*PCL-032	4	Unpaired_SNV	rs6721861	COSM442782	unknown
FAM186A	12_50744581	PCL-036*PCL-020*PCL-030	3	Unpaired_SNV	rs73108300	COSM468487	Confirmed somatic

(*Continued*)

Gene Name	Chr_Start	Samples	no. Samples	Flag	dbSNP137 rsID	COSMIC ID (v67)	Somatic Status in COSMIC website
HLA-DQA1	6_32609296	PCL-030*PCL-017	2	Unpaired_INDEL	rs9282026	COSM1443636	Previously reported
HLA-DQA1	6_32609299	PCL-030*PCL-017	2	Unpaired_INDEL	rs199556640	COSM1443637	Previously reported
IGFN1	1_201180100	PCL-027*PCL-017	2	Unpaired_SNV	rs4915223	COSM146727	unknown
IGFN1	1_201180340	PCL-027*PCL-017	2	Unpaired_SNV	rs139390045	COSM146728	unknown
KIT	4_55593464	PCL-020*PCL-030	2	Unpaired_SNV	rs3822214	COSM28026	Confirmed somatic, Previously reported
LEFTY1	1_226076669	PCL-036*PCL-030	2	Unpaired_SNV	rs41310561	COSM425442	unknown
MCF2L2	3_183056598	PCL-020*PCL-035	2	Unpaired_SNV	rs12632177	COSM1421202	unknown
MUC4	3_195506005	PCL-020*PCL-035	2	Unpaired_SNV	rs202017381	COSM1042837	Confirmed somatic
MUC4	3_195509606	PCL-020*PCL-035	2	Unpaired_SNV	rs200244334	COSM1485020	unknown
OR12D3	6_29342775	PCL-020*PCL-032	2	Unpaired_SNV	rs3749971	COSM451127	unknown
PDIA2	16_336888	PCL-027*PCL-030	2	Unpaired_INDEL	rs199887121	COSM128704	unknown
PKHD1	6_51910905	PCL-030*PCL-017	2	Unpaired_SNV	rs62406032	COSM150104	unknown
PSG2	19_43585111	PCL-030*PCL-032	2	Unpaired_SNV	rs149579909	COSM328449, COSM328448	unknown
PTTG1IP	21_46271460	PCL-020*PCL-030*PCL-032	3	Unpaired_SNV	rs2236442	COSM149244	Previously Reported
QSER1	11_32955122	PCL-036*PCL-032	2	Unpaired_SNV	rs2297781	COSM147173	Previously Reported
RETNLB	3_108475993	PCL-027*PCL-032	2	Unpaired_INDEL	rs5851607	COSM1318744	unknown
SLFN11	17_33679440	PCL-036*PCL-020	2	Unpaired_SNV	rs62079540	COSM148236	unknown
SLFN11	17_33680811	PCL-027*PCL-032	2	Unpaired_SNV	rs9898983	COSM148239	unknown

(*Continued*)

Gene Name	Chr_Start	Samples	no. Samples	Flag	dbSNP137 rsID	COSMIC ID (v67)	Somatic Status in COSMIC website
SRRM5	19_44117582	PCL-036*PCL-030*PCL-017	3	Unpaired_SNV	rs10410000	COSM439717	Confirmed somatic, Previously reported
SRRM5	19_44118188	PCL-036*PCL-030*PCL-017	3	Unpaired_SNV	rs3815422	COSM148658	Confirmed somatic, Previously reported
TNRC6B	22_40697177	PCL-036*PCL-017	2	Unpaired_INDEL	rs10617561	COSM1034397, COSM1034396	unknown
TRIM64B	11_89608899	PCL-036*PCL-027	2	Unpaired_SNV	rs201022110	COSM1475960	Confirmed Somatic, Previously Reported
ZHX2	8_123966085	PCL-036*PCL-027	2	Unpaired_SNV	rs3802264	COSM150529	unknown
ZNF598	16_2049882	PCL-020*PCL-027*PCL-035	3	Unpaired_INDEL	rs141374045	COSM307599	Confirmed somatic, Previously reported
DCP1B	12_2062323	PCL-038,PCL-032	2	Paired_INDEL, Unpaired_INDEL	rs149912567	COSM249162	Confirmed Somatic, Previously Reported
IFT122	3_129214358	PCL-016,PCL-035	2	Paired_SNV, Unpaired_SNV	rs73204230	COSM1418827	Previously Reported
VN1R4	19_53770746	PCL-026,PCL-017	2	Paired_SNV, Unpaired_SNV	rs140031028	COSM130160	Previously Reported

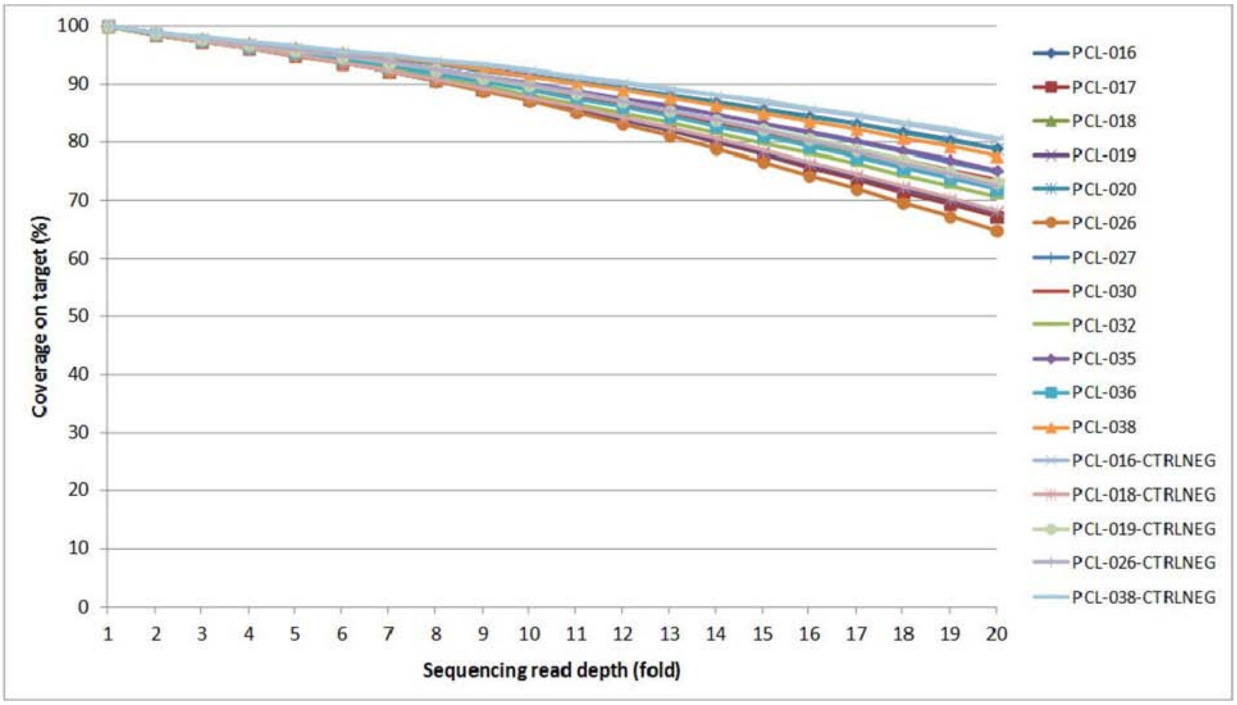

Supplementary Figure S1: On target coverage in relation to read depth. For each sample, mean coverage on target regions (totally 62 Mb) is plotted in relation to sequencing read depth. At 10x depth, all samples had at least 85% of target regions covered.

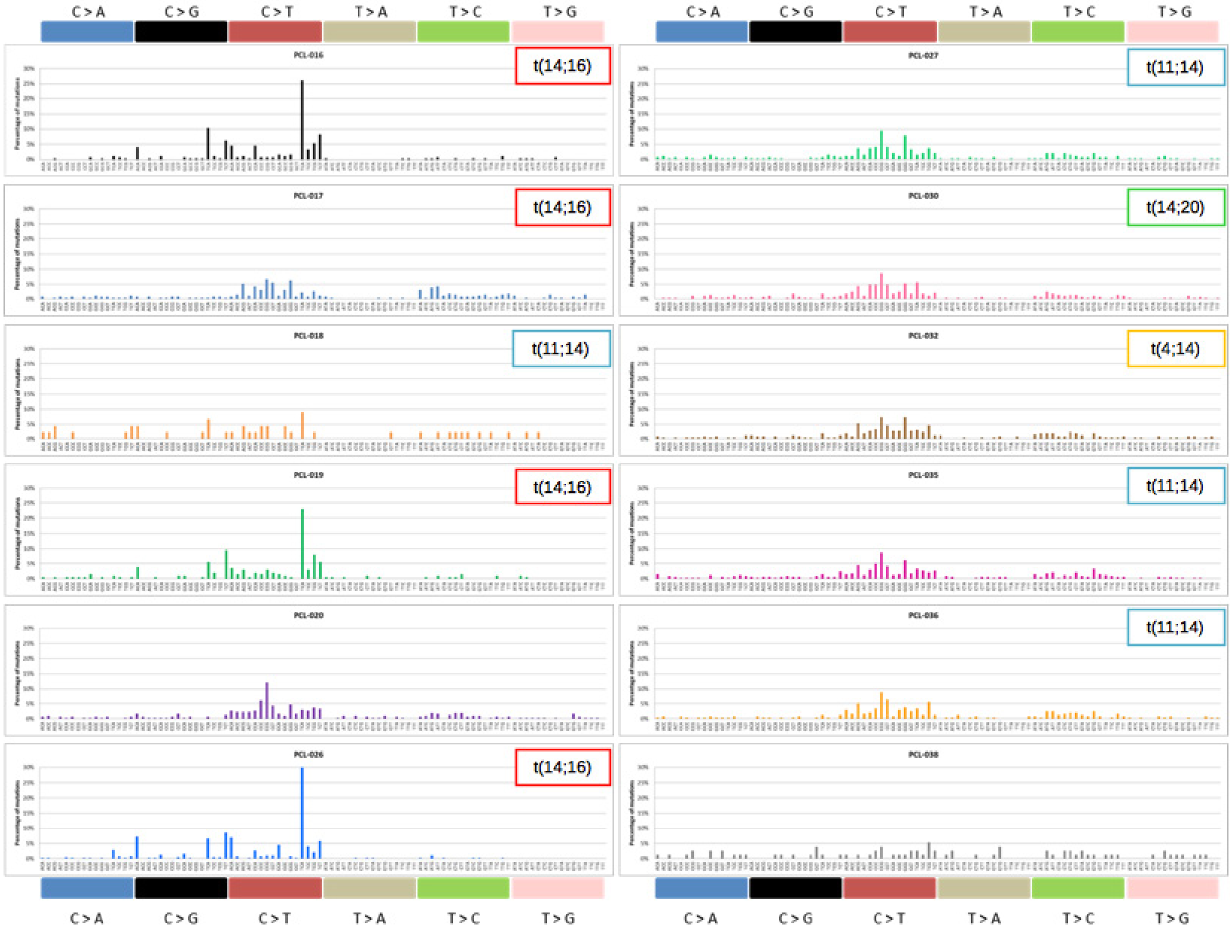

Supplementary Figure S2: Mutational signatures for individual samples. Coding somatic SNVs (both syn and non-syn) identified in each of the 12 pPCL samples were classified according to nucleotide change and sequence context into 96 possible mutated trinucleotides, whose relative contribution is represented for each sample. Substitutions are displayed along the horizontal axes and percentages of mutations attributed to each type are shown on the vertical axes. Translocations carried by each sample are reported as assessed by FISH.

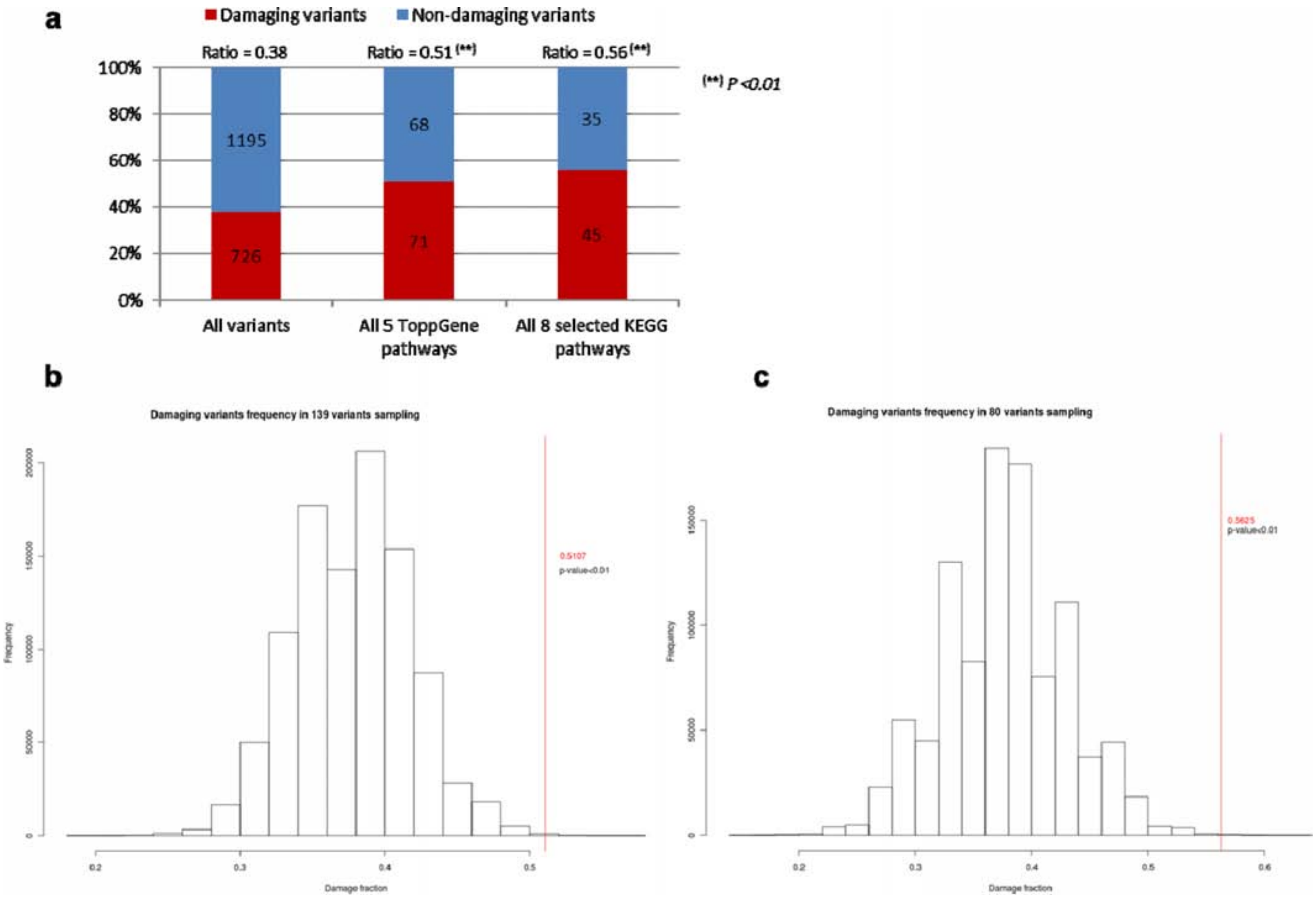

Supplementary Figure S3: Resampling tests for damaging variants. A. The ratio between damaging and non-damaging variants was calculated for the overall list of 1, 928 coding somatic non-silent variants (used as background), and then for variants found in the five significantly enriched ToppGene pathways, and in the eight selected pathways. Variants were considered as damaging if they were SNVs predicted as deleterious by at least three out of five prediction algorithms included in Annovar tool, or frameshifting indels. Fisher exact test was applied to calculate enrichment with respect to background ratio (***p*-value < 0.01). **B.** By performing random resampling (1, 000, 000 samplings) of 139 variants in our dataset, we obtained the damaging ratio distribution plot and calculated the probability of having the same fraction of damaging variants as that found in the five significantly enriched ToppGene pathways. Red vertical bar indicates the damaging ratio calculated for the five pathways (*p*-value < 0.01). **C.** By performing random resampling (1, 000, 000 samplings) of 80 variants in our dataset, we obtained the damaging ratio distribution plot and calculated the probability of having the same fraction of damaging variants as that found in the eight selected pathways. Red vertical bar indicates the damaging ratio calculated for the eight pathways (*p*-value < 0.01).
